# Spatio-temporal changes in clusters of gastric cancer incidence: The impact of nationwide cancer control programs in South Korea

**DOI:** 10.1371/journal.pone.0349384

**Published:** 2026-06-16

**Authors:** Ji Hyun Kim, Won Kyung Kim, Jeongseon Kim, Sun-Young Kim

**Affiliations:** National Cancer Center Graduate School of Cancer Science and Policy, National Cancer Center, 323 Ilsan-ro, Ilsandong-gu, Goyang-si, Gyeonggi-do, Republic of Korea; Sichuan University, CHINA

## Abstract

**Background:**

Gastric cancer (GC) is a global health concern, with incidence and mortality varying significantly across populations. The spatial heterogeneity of GC may reflect differences in prevention strategies and their effectiveness, highlighting the need to identify high-risk areas and their possible contributing factors.

**Objective:**

Leveraging the decline of GC incidence since 2012 affected by nationwide cancer prevention programs in South Korea, this study aimed to identify high- and low-risk clusters and explore the changes in spatial clusters of GC incidence between 2009–2013 and 2014–2018 before and after the decline, respectively. In addition, we identified geographic characteristics associated with cluster.

**Method:**

Using national cancer registry and geographic data across 243 districts, we performed spatial clustering analyses in each period. We identified the high- and low-risk areas as the overlapping clusters by two common clustering approaches of local Moran’s I and Getis Ord Gi*. Then, we compared 23 geographic characteristics between high- and low-risk areas and two periods.

**Results:**

The average GC incidence rate declined in both high- (96–79 per 100,000 people) and low-risk districts (73–61) between 2009–2013 and 2014–2018. While cluster locations remained stable, low-risk districts expanded notably (26–37) and high-risk districts slightly reduced (31–28). As opposed to little geographic characteristics that showed the significant difference between high- and low-risk areas in the early period, the later period gave large green space, frequent regular walking, and reduced self-related obesity in the low-risk area, in addition to sociodemographic advantages, compared to those in the high-risk area (p-value <0.002).

**Conclusion:**

Our findings suggest the potential effectiveness of lifestyle- and/or environment-focused prevention for GC incidence and the need of locally-tailored strategies to reduce cancer burden.

## 1. Introduction

Gastric cancer (GC) remains a major health issue, with approximately one million new cases and 0.7 million deaths as the fifth position of both incidence and mortality worldwide [1]. Although overall incidence and mortality rates of GC have been steadily declining in most countries [2], there were considerable variations depending on the region and subpopulation. For example, 10 countries that showed the increase in estimated annual percent change of age-standardized GC incidence for 1990–2017 were mostly African nations [2,3]. Apart from decreased or stabilized incidence rates among those aged 50 years or older, the younger population less than 50 showed increased rates in 15 countries including the U.K., the Netherlands, Belarus, Canada, and Chile [4]. Despite relatively low incidence and mortality of GC compared to other cancer types, the survival is generally poor in most countries, with 20–40% of 5-year age-standardized net survival [5].

GC is known as one of the preventable cancer types, indicating a potential to accomplish the success of various interventive efforts. The Organization for Economic Cooperation and Development classified GC as a disease of preventable mortality, which can be avoidable by primary preventive interventions prior to disease onset [6]. The much higher survival rate of over 60%, despite the high incidence, maintained in several East Asian countries compared to other countries may also suggest the significant role of cancer prevention for GC [7]. For example, South Korea and Japan established national GC screening programs including upper gastrointestinal series or endoscopy in 2002 and 2016, respectively, for adults aged 40 and over. The proportion of surgically treated early GC doubled from 29% in 1995 to 64% in 2019 in South Korea [8]. In addition, as GC is largely affected by behavioral characteristics [9], prevention activities that encourage smoking and drinking cessation, low salt intake, normal body weight, and regular physical activity might have resulted in gradual incidence decline in South Korea [10,11].

The preventive potential of GC can be accelerated when we target high-risk areas of GC and identify risk factors in such areas. GC incidence showed the geographic patterns across countries as well as within a country [12,13]. Spatial clustering can help identify high-risk areas and implement prevention programs to reduce incidence [14]. Furthermore, clusters could vary over time, due to the change in sociodemographic characteristics as well as the progression of cancer prevention strategies [15–17]. The relationships between the temporal changes of spatial clusters and characteristics in corresponding areas can guide us to identify effective preventive activities.

This study aims to identify spatial clusters of GC incidence across 243 districts in South Korea, and to investigate geographic characteristics relevant to clusters. In addition, we compared geographic characteristics between high- and low-risk clusters and between the two time periods of 2009–2013 and 2014–2018. We chose these two periods based on the data availability of cancer incidence. The high GC incidence as well as the distinctive decline after the implementation of nationwide cancer control programs in South Korea can provide a good opportunity to investigate effective preventive intervention associated with GC incidence decline. In South Korea, GC remains one of the most common cancers, with 29,487 new cases and the age-standardized incidence rate of 26.8 per 100,000 people in 2022 [18]. The age-standardized prevalence reached 302.8 for 100,000 people, and the 5-year relative survival rate was 78.4% in 2018–2022. After the establishment of the South Korean National Cancer Control Plan in 1996, GC incidence increased until 2012 but began to decline afterwards [11]. We hypothesized that this change could be due to the impact of the first and second national cancer control programs carried out over 20 years for 1996–2015 and the difference in decline of GC incidence across districts could be attributed to different preventive features across districts.

## 2. Materials and methods

### 2.1. Data for GC incidence and geographic characteristics

We obtained 5-year age-standardized incidence rates of GC across 249–252 districts (“Si-Gun-Gus”) for 2009–2013 and 2014–2018. This statistics is a type of the Cancer Registration Statistics (CRS) created from the Korea Central Cancer Registry (KCCR) and are available in the South Korean Statistical Information Service (KOSIS, https://kosis.kr/) [19]. District-specific age-standardized incidence rates are calculated for 24 types of cancer over five-year periods, with age standardization by using the administrative population as of midyear in July 1, 2000. The district is the second level of the administrative divisions in South Korea. Three hierarchical structures of administrative division include 17 metropolitan cities and provinces (“Si-Do”; Population range: 624,395–12,522,606), 252 districts (“Si-Gun-Gu”; 10,153–660,302), and 3,554 neighborhoods (“Eup-Myeon-Dong”; 116–96,266) in 2015. Given small numbers of new cancer cases in some districts resulting in confidentiality concerns, district-level incidence rates are available as 5-year aggregation. GC incidence was defined according to the International Classification of Diseases 10th edition (ICD–10) code of “C16”. All cancer cases in South Korea were originally registered according to the International Classification of Diseases for Oncology, 3rd edition (ICD-O–3) and subsequently converted to the ICD–10 [18].

We also obtained 23 district-specific characteristics which are known as being associated with GC in previous studies (S1 Table). These geographic characteristics represent eight categories of demography, socioeconomic status, lifestyle, medical status, healthcare infrastructure, medical accessibility, health screening, and physical environments (S2 Table and S2 Text). Previous studies of spatial regression showed that GC incidence was positively associated with large elderly population, socioeconomic deprivation, unhealthy behaviors, and/or limited healthcare access. We downloaded district-specific proportions and summaries of demography, socio-economic status, GC screening participation, and healthcare infrastructure from the KOSIS in 2010 and 2015 at the middle of two 5-year periods of cancer incidence. GC screening rate was the proportion of individuals aged ≥ 40 years who underwent GC screening either gastrointestinal series or endoscopy among the eligible population in a given year. District-level lifestyle, medical status, medical accessibility, health screening, and physical environments are available in the Korea Community Health Survey (KCHS) website (https://chs.kdca.go.kr/).

Because district boundaries had changed due to the consolidation or separation of districts over the 10-year study period, we applied a uniform boundary based on the year 2015 that is available in the Statistical Geographic Information Service (https://sgis.kostat.go.kr/). Specifically, we combined or split the districts that do not retain the same boundaries to those in 2015, and recomputed cancer incidence rates and geographic characteristics for modified districts. While we computed population-weighted averages for combined districts, we assigned the same proportions as the original or the counts multiplied by population or area fraction for separated districts. In addition, we excluded nine districts which are islands or located on the coastline without neighbors, resulting in 243 districts for our spatial clustering analyses.

This study used anonymized, aggregated public data, and ethical review was waived by the Institutional Review Board of the National Cancer Center (IRB number: NCC2025–0361).

### 2.2. Study design and period rationale

This ecological study examined spatial clusters of gastric cancer incidence and their relationships with geographic characteristics across 243 districts in South Korea. The study periods for 2009–2013 and 2014–2018 were determined based on the availability of district-level cancer incidence data aggregated into 5-year intervals. These periods also correspond to phases before and after the observed decline in gastric cancer incidence around 2012, allowing us to explore temporal and spatial differences in incidence patterns. District-level geographic characteristics were applied as ecological proxies to represent population-level exposures. We used geographic characteristics in 2010 and 2015 as the mid-year of two study periods based on data availability.

### 2.3. Clustering analysis

We assessed the spatial clusters of age-standardized GC incidence rate across districts by applying two commonly applied clustering analysis methods: local Moran’s I and Getis-Ord-Gi*. While local Moran’s I identifies spatial clusters based on significantly similar or dissimilar values between a target and neighboring areas, Getis-Ord Gi* relies on the spatial concentration of significantly high or low values in a target and neighboring areas. We identified the high- and low-risk areas of GC incidence as the overlapping districts clustered as high- and low-risk areas from two approaches, as suggested in previous studies [20,21].

The detailed description of two approaches is provided in the “Details of statistical methods to assess spatial autocorrelation and spatial clusters” of the Supplementary Materials **(S1 Text)**. In brief, the local Moran’s I method defines the spatial cluster by using local Moran’s I statistic computed as the spatial autocorrelation of GC incidence in each target district with those in neighboring districts [22]. Here, we used the Queen’s contiguity method to identify neighboring districts which share either edges or corners with a target district [23]. Specifically, spatial weights of 1 and 0 were assigned to neighboring and all the other districts, respectively, and row-standardized to make each row sum of spatial weight matrix equal to 1. We chose the Queen’s method, instead of the distance-based method, to effectively define neighboring districts given highly irregular shapes and sizes of districts in South Korea (minimum and maximum area size: 3 and 1,815 km^2^, respectively, in 2015). This neighborhood definition was commonly applied in epidemiological studies of environments [24]. The districts with significantly high positive z-scores of local Moran’s I statistics imply a cluster where a target district has similarly high or low GC incidence as its neighborhood. These clustered areas include high-high and low-low clusters, indicating districts with high and low GC incidence, respectively, in target and neighboring districts compared to the overall mean. On the other hand, the Getis-Ord-Gi* approach assesses the local sum of weighted GC incidence for each target district together with the neighboring districts and compares to the sum of weighted GC incidence for all districts [25]. As Getis-Ord-Gi* statistics are considered as equivalent to z scores, the districts with significantly positive and negative z-scores, defined as hot and cold spots, indicate the clusters with higher and lower GC incidence than would be expected. Finally, we defined the “high-risk area” as the overlapping districts of the high-high cluster identified by the local Moran’s I and the hot-spot identified by the Getis-Ord-Gi* [20,21]. Likewise, the “low-risk area” indicates the overlapping districts of the low-low cluster and the cold-spot.

Furthermore, we examined the sensitivity of our cluster detection to neighborhood definition and inferential criteria. Three alternative spatial weight specifications included Rook contiguity, k-nearest neighbors, and distance band approaches (S3 Table). For k-nearest neighbors, we applied seven neighbors that gave the maximum global Moran’s I statistic beyond the average contiguous neighbors of five (S1 Fig). The distance band of 35 kilometers was chosen based on a previous study of GC clustering analysis in South Korea [26]. K-nearest neighbors also allowed the clustering analysis using all 252 districts without excluding nine districts as islands or on the coastline. In addition, as conservative inferential criteria, we applied permutation-based inference with a significance threshold of p-value < 0.05 and Benjamini–Hochberg false discovery rate with a threshold q-value < 0.10.

### 2.4. Analysis for geographic characteristics and GC clusters

We examined the difference in each geographic characteristic between high-and low-risk areas using Student t-test or Mann Whitney U test in each period. The statistical significance was defined as p-value < 0.00217 (i.e., 0.05/23 geographic variables) with multiple comparison adjustment. In addition to the absolute mean difference, we computed the average relative difference as the difference in averages of each geographic characteristic between high- and low-risk areas divided by the average in the low-risk area. Then, to examine the differences in relevant geographic characteristics between high- and low-risk areas and between the two periods, we computed the differences of the average absolute and relative differences between the two periods. We also presented the standardized mean difference (SMD) that is the average difference divided by pooled standard deviation to compare the difference by minimizing the impact of the spatial correlation of geographic characteristics (S3 Text) [27]. In addition, we applied an interaction model including the status of clusters and periods, and evaluated the statistical significance in the differences of characteristics between high- and low-risk areas in the later period compared to the early period.

In order to examine the robustness of our findings for geographic characteristics and GC incidence, we performed three sensitivity analyses. First, given the long latency of gastric carcinogenesis, we applied geographic characteristics in the early period for 2010–2011 to the incidence in the later period for 2014–2018. Second, we used age-specific GC incidence rates, instead of the binary indicator of high- vs. low-risk areas, and conducted regression analyses on each geographic characteristics within each period as well as for their differences between periods. Lastly, we explored the role of geographic characteristics in the four types of districts where risk cluster status changed over the two periods: high-risk to non-significant (NS), NS to low-risk, low-risk to NS, and NS to high-risk areas. There were no districts that showed cluster shifts of high- to low-risk or low- to high-risk areas.

Data processing and statistical analyses were carried out in ArcGIS 10.5 (ESRI, Redland, CA, USA), R 4.4.1, (R Core Team, Vienna, Austria), and SAS 9.4 (SAS Institute Inc., Cary, NC, USA).

## 3. Results

### 3.1. Data exploration for GC incidence and geographic characteristics

**Fig 1:**
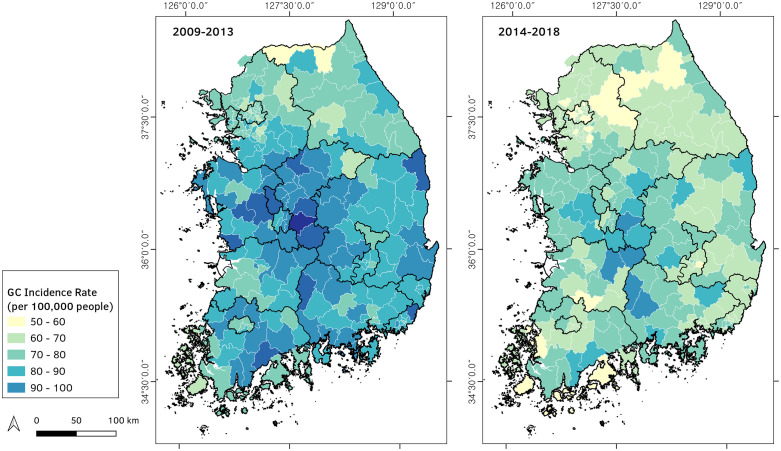
Maps for age-standardized incidence rates of gastric cancer across 243 districts in South Korea for 2009–2013 and 2014–2018.

The median 5-year age-standardized incidence rate for GC across 243 districts decreased from 83.4 to 68.8 per 100,000 people between 2009–2013 and 2014–2018. The districts that showed high GC incidence slightly changed between the two periods: the incidence was high in the districts located in the central inland region of South Korea for 2009–2013 and in Southern inland region for 2014–2018 (**Fig 1**). Both periods gave significant spatial autocorrelation of GC incidence across districts (global Moran’s I statistic = 0.52 and 0.47 in 2009–2013 and 2014–2018, respectively). Most district-specific characteristics showed significant changes between the two periods (**Table 1**). From 2009–2013–2014–2018, the average district-specific proportions of older adults aged ≥ 65 years, urban dwellers, and the proportion of individuals with higher educational attainment increased by 2.4, 1.8, and 5.0%, respectively. The proportion of current smokers declined, whereas the proportion of heavy drinkers increased. Although fewer people eat breakfast, low-salt preference became more common. While medical accessibility and health screening rates including GC screening participation improved, the prevalence of chronic diseases such as hypertension, diabetes, and dyslipidemia also increased.

**Table 1:** Means and standard deviation of 23 geographic characteristics across 243 districts between 2009–2013 and 2014–2018.

Geographic characteristics	2009–2013	2014–2018	Direction of change between two periods
**Demography**			
% of older adults ≥ 65 years	14.9 ± 7.3	17.3 ± 7.7	+^a^
Sex ratio	100.7 ± 4.1	100.5 ± 4.9	–
Population density	43.5 ± 64.8	42.7 ± 62.9	-^a^
% of urban-dwelling population	75.3 ± 27.5	77.1 ± 26.3	+^a^
**Socio-economic status**			
Growth regional domestic product per capita (1,000 USD /person)	19.2 ± 18.9	24.4 ± 22.7	+ ^a^
% of higher education	25.6 ± 10.5	30.4 ± 10.3	+ ^a^
**Lifestyle**			
% of breakfast ≥ 5 times/week	73.4 ± 5.3	66.9 ± 5.6	-^a^
% of low-salt preference^b^	9.8 ± 2.7	11.1 ± 2.4	+ ^a^
% of current smoking	25.3 ± 2.7	22.1 ± 2.7	-^a^
% of heavy drinking	14.7 ± 4.0	19.0 ± 3.4	+ ^a^
% of moderate to vigorous physical activity	23.8 ± 9.0	23.9 ± 7.1	–
% of regular walking	43.2 ± 12.1	41.0 ± 11.6	–
% of self-reported obesity	22.6 ± 2.7	26.4 ± 2.7	+ ^a^
**Medical status**			
% of doctor’s diagnosis of hypertension	14.5 ± 1.8	15.3 ± 1.8	+ ^a^
% of doctor’s diagnosis of diabetes	5.6 ± 0.9	6.1 ± 1.0	+ ^a^
% of doctor’s diagnosis of hyperlipidemia	7.9 ± 2.3	10.1 ± 2.3	+ ^a^
**Healthcare infrastructure**			
Number of hospital beds per 1000 people	11.4 ± 6.7	14.5 ± 8.9	+ ^a^
Number of medical personnel per 1000 people	2.3 ± 2.0	2.5 ± 2.2	+ ^a^
**Medical accessibility**			
% of unmet healthcare needs	13.6 ± 4.2	12.2 ± 3.7	- ^a^
**Health screening**			
% of cancer screening examinees for the previous 2 years	43.1 ± 4.6	48.5 ± 5.5	+ ^a^
% of gastric cancer screening examinees	45.9 ± 4.3	58.4 ± 4.5	+ ^a^
% of health screening examinees for the previous 2 years	56.2 ± 4.9	62.4 ± 4.6	+ ^a^
**Physical environment**			
% of urban forest coverage within residential area	3.0 ± 6.4	3.9 ± 8.0	+

^a^Statistical significance (p-value < 0.00217 [0.05/23]) for each variable in paired t-test (all variables in lifestyle, medical status, medical accessibility, and health screening categories and the number of hospital beds in healthcare infrastructure) or Wilcoxon signed-rank test (all variables in demography, socioeconomic status, and physical environment categories and the number of medical personnels in healthcare infrastructure).

^b^elf-reported low-salt preference by 1) taking low levels of salt intake, 2) not adding salt or soy sauce to the dishes served on tables; and 3) not dipping to soy sauce for fried foods.

### 3.2. Spatial clusters of GC incidence rates between two time periods

**Fig 2:**
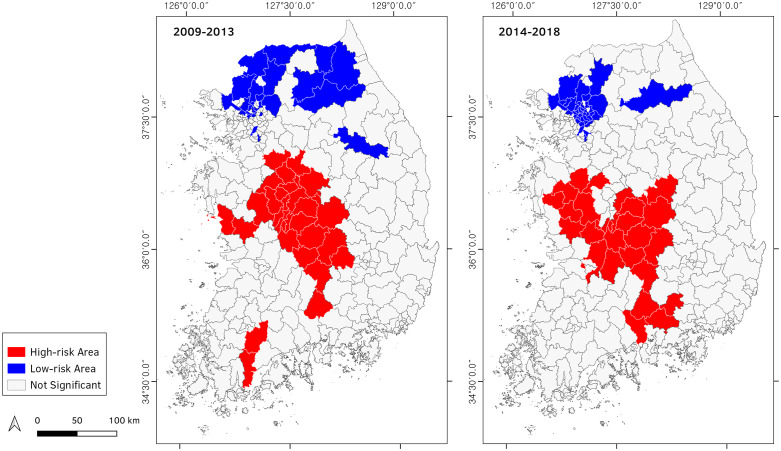
Maps of high- and low-risk areas of age-standardized incidence rates for gastric cancer across 243 districts in South Korea for 2009–2013 and 2014–2018.

**S2 Fig** shows the locations of spatial clusters for GC incidence defined by local Moran’s I and Getis-Ord-Gi*. Both methods identified similar districts as clusters: high-high and hot-spot clusters in the central inland, and low-low and cold spot clusters in the northern region. These similar cluster locations between the two approaches gave high- and low-risk areas in the central and northern regions, respectively (**Fig 2**). The locations of the two types of clusters were generally consistent between the two time periods. The low-risk area was expanded from 26 districts for 2009–2013 to 37 districts for 2014–2018, while the high-risk area was slightly reduced from 31 to 28 districts. The average age-standardized incidence rates for GC were 95.9 and 73.4 per 100,000 people in high- and low-risk areas, respectively, for 2009–2013. These rates decreased in both high- and low-risk areas to 79.2 and 61.1 per 100,000 people for 2014–2018 (**Table 2**). The locations of high- and low-risk areas were largely consistent in our sensitivity analyses using alternative spatial weight specifications and inferential criteria (S1, S3, and S4 Figs).

**Table 2:** Geographic characteristics in high- and low-risk areas and their differences by 2009–2013 and 2014–2018.

Geographic characteristics	2009–2013			2014–2018		
	High-risk area (N = 31) ^c^	Low-risk area (26)	HL difference^d^	High-risk area (28)	Low-risk area (37)	HL difference^d^
	Mean (SD)	Mean (SD)	AAD^a^	Mean (SD)	Mean (SD)	AAD^a^
**Gastric cancer incidence rate**						
Age-standardized incidence rate per 100,000	95.9 (6.0)	73.4 (6.9)	22.5^b^	79.2 (7.0)	61.1 (3.1)	18.1^b^
**Demography**						
% of older adults ≥ 65 years	17.5 (7.3)	12.1 (4.0)	5.4	22.3 (7.5)	12.5 (2.6)	9.8^b^
Sex ratio	101.0 (3.6)	101.7 (4.6)	–0.7	99.3 (2.9)	98.3 (3.9)	1.0
Population density	7.5 (12.8)	68.4(76.3)	–60.9	5.6 (12.1)	105.0 (75.8)	–99.4^b^
% of urban-dwelling population	64.7 (25.5)	84.8 (20.8)	–20.1	57.7 (24.6)	95.9 (11.5)	–38.2^b^
**Socio-economic status**						
Growth regional domestic product per capita (1,000 USD/person)	17.7 (7.2)	27.1 (46.8)	–9.4	21.5 (9.2)	31.4 (49.2)	–9.9
% of higher education	21.3 (8.9)	30.8 (9.7)	–9.5^b^	24.6 (8.5)	42.4 (10.2)	–17.8^b^
**Lifestyle**						
% of breakfast ≥5 times/week	73.5 (4.7)	71.3 (3.9)	2.2	69.5 (5.8)	63.5 (3.9)	6.0^b^
% of low-salt preference	9.7 (2.4)	9.4 (2.1)	0.3	10.8 (2.7)	12.3 (1.9)	–1.5
% of current smoking	25.9 (2.5)	25.2 (2.3)	0.7	21.7 (2.6)	21.1 (3.0)	0.6
% of heavy drinking	14.1 (3.6)	15.6 (3.2)	–1.5^b^	19.3 (3.5)	17.9 (2.7)	1.4
% of moderate to vigorous physical activity	22.4 (4.6)	23.0 (8.2)	–0.6	24.2 (6.9)	21.8 (3.4)	2.4
% of regular walking	39.2 (10.7)	48.7 (11.8)	–9.5	39.7 (10.2)	50.0 (10.2)	–10.3^b^
% of self-reported obesity	22.1 (3.2)	24.0 (3.1)	–1.9	26.2(2.8)	24.9 (1.9)	1.3^b^
**Medical status**						
% of doctor’s diagnosis of hypertension	14.3 (2.0)	15.6 (1.2)	–1.3	15.6 (2.0)	15.2 (1.5)	0.4
% of doctor’s diagnosis of diabetes	5.5 (1.0)	5.9 (0.8)	–0.4	6.1 (0.7)	5.8 (1.0)	0.3
% of doctor’s diagnosis of dyslipidemia	7.3 (1.9)	9.2 (1.9)	–1.9^b^	10.1 (2.5)	11.4 (1.4)	–1.3
**Healthcare infrastructure**						
Number of hospital beds per 1000 people	12.5 (7.7)	8.6 (3.8)	3.9	14.6 (6.5)	9.5 (4.2)	5.1
Number of medical personnel per 1000 people	2.0 (0.8)	2.8 (3.0)	–0.8	2.2 (0.8)	3.6 (3.3)	–1.4
**Medical accessibility**						
% of unmet healthcare needs	12.3 (4.7)	14.0 (3.2)	–1.7	12.6 (4.5)	11.3 (2.5)	1.3
**Health screening**						
% of cancer screening examinees for the previous 2 years	44.7 (3.3)	44.0 (2.7)	0.7	50.0 (5.9)	50.4 (5.0)	–0.4
% of gastric cancer screening examinees	50.0 (3.4)	44.4 (3.5)	5.6^b^	61.3 (3.6)	54.6 (2.9)	6.7^b^
% of health screening examinees for the previous 2 years	55.3 (4.6)	57.3 (4.0)	–2.0	61.3 (5.8)	63.3 (4.1)	–2.0
**Physical environment**						
% of urban forest coverage within residential area	1.5 (1.6)	2.9 (3.3)	–1.4	1.7 (1.8)	6.9 (11.5)	–5.2^b^

^a^Average absolute difference in characteristics between high- and low-risk areas calculated as (average of high − low) for each period.

^b^Statistical significance assessed by comparing high- and low-risk areas within each period using Student’s t-test (all variables in lifestyle, medical status, medical accessibility, and health screening categories and the number of hospital beds in healthcare infrastructure) or the Mann–Whitney U test (all variables in demography, socioeconomic status, and physical environment categories and the number of medical personnels in healthcare infrastructure), with a Bonferroni-corrected significance threshold of p < 0.00217 (0.05/23).

^c^Number of districts

^d^Difference between low- and high-risk areas

### 3.3. Geographic characteristics between GC clusters and time periods

**Table 2**, **S4 Table****, and Fig 3** present the comparison of district-level geographic characteristics between the high- and low-risk areas as well as between the two periods. In the earlier period for 2009–2013, the high-risk area showed significantly lower proportions of educational attainment and dyslipidemia diagnosis and higher proportion of GC screening participation than those in the low-risk area. In the later period for 2014–2018, educational attainment and GC screening gave the consistent pattern of significantly lower and higher proportions, respectively, in the high-risk area. In addition, demographic, lifestyle, and environmental characteristics became significantly different between high- and low-risk areas (Table 2 and S4 Table). The high-risk area showed the higher proportion of older adults, lower population density, and the lower proportion of urban-dwelling population. While proportions of regular walking and urban forest coverage within residential areas were lower, the proportions of self-reported obesity and frequent breakfast consumption were higher.

**Fig 3:**
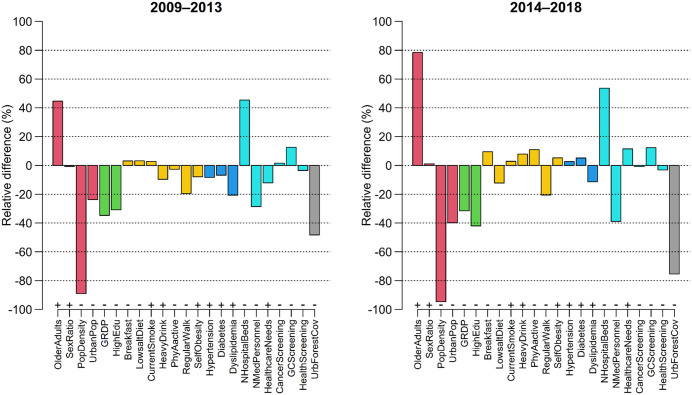
Average relative differences in geographic characteristics between high- and low-risk areas by 2009–2013 and 2014–2018. Relative differences were calculated as (high-risk − low-risk) / low-risk × 100. Positive and negative relative differences indicate higher levels in high- and low-risk areas, respectively. The ‘+’ and ‘−’ signs on x-axes indicate anticipated directions in the association with GC incidence.

When we compared absolute and relative differences in high- vs. low-risk areas between the two periods, the patterns of positive and negative differences in demographic characteristics were generally similar, but the quantity of differences were more pronounced for 2014–2018 than for 2009–2013 (S4 Table and Fig 3). The positive difference in older adults and negative difference in urban-dwelling population and urban forest coverage within the residential areas, indicating higher and lower proportions in the high-risk area, were greater with 16–33% changes (relative difference: 45% for 2009–2013 to 78% for 2014–2018, –24 to –40%, and –48 to –75%, respectively). Interestingly, some behavioral characteristics showed the reverse direction between the two periods from less to more healthy patterns in the low-risk area. The positive difference for low-salt preference and the negative difference for heavy drinking in the early period became negative and positive differences in the later period. Sex ratio, hypertension diagnosis, and unmet healthcare needs also gave the reverse patterns between the two periods. Although GC screening showed higher rates in high-risk areas in both periods, these differences were consistent between two periods, resulting in a little change (–0.3%). The interaction analysis by risk area and period also showed no significant interaction for GC screening. The patterns of absolute and relative differences were consistent in SMDs.

In our sensitivity analysis using geographic characteristics prior to GC incidence, the patterns were overall consistent with mostly < 5% relative differences compared to those in the original analysis using later-period geographic characteristics (S5 Table). Regression analyses using district-level GC incidence rates also showed the negative and positive associations with educational attainment and GC screening, respectively, in both periods (S6 Table). When we used the differences in geographic characteristics and GC incidence between two periods, the negative association of educational attainment remained. In contrast, the association with GC screening participation disappeared. For four types of districts where risk cluster status changed over the two periods, 15 and 17 districts of high-risk and NS areas for 2009–2013 became NS and low-risk areas, respectively, indicating decreased risks of GC incidence. In contrast, 6 and 12 districts in low-risk and NS areas changed to NS and high-risk areas, respectively. However, these districts did not show significant differences in geographic characteristics between the two periods (S5 and S6 Figs).

## 4. Discussion

The present study assessed the changes in the spatial clusters of GC incidence and associated geographic risk factors in South Korea between two 5-year periods before and after age-standardized GC incidence began to decrease in 2012 as the nationwide cancer control efforts were expanded. Compared to the early period for 2009–2013, the low-risk area was expanded, and high-risk area was reduced in the recent period for 2014–2018, although the high-risk area remained in central regions. The high-risk area showed older population, socioeconomic deprivation, less healthy behavior, and limited green space, particularly during the 2014–2018 period.

Our findings of cluster analyses can suggest the effectiveness of cancer prevention efforts. In South Korea, GC incidence remained the highest compared to other major cancer sites until 2011 even after the nationwide cancer prevention program were established in 1996. The incidence began to decline afterwards with a particularly sharp decrease by 5.3% from 2012 to 2015 [10]. The consistently high incidence in the early period of the cancer prevention program could be due to the active implementation of and increased participation in cancer screening [28,29]. Between 2009–2013 and 2014–2018, median district-specific GC incidence decreased from 83.4 to 68.8 new cases per 100,000 people. The low-risk area expanded from 26 to 37 districts, while the high-risk area slightly reduced from 31 to 28 districts. The enlarged low-risk area and shrinking high-risk area may be partially explained by the implementation of cancer prevention guidelines from the second-term National Cancer Control Plan applied for 2006–2015. This effort particularly targeted behavioral risk factors and led to the increase in smoking cessation, intake of fruits and vegetables, and cancer screening rates [11,30].

The changes in major behavioral factors associated with cancer between high- and low-risk areas during 2014–2018 compared to 2009–2013 may reflect the changes in population-level behaviors over time derived by targeted cancer prevention efforts. Major behavioral characteristics either significantly differed between high- vs. low-risk areas or the difference became wider only in the recent period. The proportions of regular walking and self-reported obesity were not statistically different between high- and low-risk areas in the early period, but became significantly higher and lower, respectively, in the low-risk area in the recent period. The proportion of low-salt preference was slightly lower, and the proportion of heavy drinking was higher in the low-risk area during the early period, but these patterns were reversed during the recent period. A systematic review also shows that regular physical activity has a protective effect against GC (relative risk 0.83; 95% CI, 0.76–0.91), possibly due to improved insulin resistance and anti-inflammatory or anti-tumorigenic responses [31]. Excessive salt may elevate intra-gastric sodium concentration and act as an irritant, damaging the stomach mucosal barrier [32,33]. A global meta-analysis identified a higher intake of salt-preserved foods as a probable risk factor of GC incidence [34]. A population-based cohort study in South Korea also showed an increased GC risk associated with salt preference [35]. The northern inland region including Seoul, Gyeonggi-do, and Gangwon-do identified as the low-risk area in our study showed higher preference of low-salt diet [36].

Sociodemographic and environmental factors were significantly less favorable in the high-risk area consistently over the entire period. Aging population, rural population, and low educational attainment indicating sociodemographic deprivation were largely prevalent in the high-risk area. Previous spatial analyses in South Korea also reported high GC incidence in the areas with socioeconomic deprivation using employment, occupation, and healthcare infrastructure in addition to education and population characteristics [17,37]. These social indicators are also possibly related to behavior patterns that directly result in substantial differences in cancer outcomes [38]. In addition, our study showed predominantly low residential urban forest coverage in the high-risk area. Low green space in residences could lead to limited physical activity and/or represents high air pollution that could affect cancer incidence [39,40].

Some of our findings are contradictory to previous findings or expected patterns. Previous epidemiological studies showed that skipping breakfast was associated with impaired glucose metabolism, chronic inflammation, and cancer risk including GC [41,42]. In contrast, we found a significantly higher proportion of regular breakfast in the high-risk area than the low-risk area. This could be because the high-risk area includes a large portion of elderly population who tends to consume their meals earlier in a day [43]. We also hypothesized that cancer screening may have affected spatial differences in GC incidence. Although we did not find the difference in cancer screening, GC screening participation was consistently higher in the high-risk area than in the low-risk area in both periods. However, these differences were not statistically different between the two periods, suggesting a large contribution of lifestyle and/or environmental exposures to temporal changes in GC incidence compared to screening programs.

Our study did not consider *H. pylori* that was well addressed for its association with GC incidence and classified as a group I carcinogen, because district-level infection or eradication rates of *H. pylori* are unavailable. Previous South Korean studies reported high *H. pylori* infection among older adults and those with low income or low educational attainment [44,45]. Our finding of the higher proportion of older adults and lower proportion of high school graduates in the high-risk area than in the low-risk area suggests the partial contribution of *H. pylori* to the distinction between high- and low-risk areas. In addition, a provincial-level analysis showed low prevalence and high eradication rates in Seoul, Gyeonggi, and Gangwon provinces which consist of all districts of the low-risk area in our study (S7 Fig). Although it is less likely that *H. pylori* alone fully accounts for the geographic disparities of GC incidence given the substantial decline of *H. pylori* seroprevalence in South Korea over recent decades from 67% in 1998 to 44% in 2016–2017, the impact of unmeasured *H. pylori* burden cannot be excluded [45].

This study has several limitations that provide future research topics. The consumption of specific food such as fruits, vegetables, and sodium was related to GC prevention or development [32]. Detailed information on the amount of intake can help clarify the impact of such dietary patterns. In addition, we did not examine the cluster locations of GC incidence by histological subtypes of GC which may also be related to different risk factors, because of data unavailability. Future studies should re-examine the spatial patterns and risk factors by GC subtypes. Our results also could have been affected by biased responses in self-reported survey and temporal ambiguity in aggregated incidence data. We assessed geographic characteristics from single representative midpoint years with the assumption of relatively stable temporal patterns and reasonable representativeness for each corresponding period. However, these years corresponding to census- and survey-based data collection cycles may not fully capture year-to-year variability within each 5-year incidence period. Furthermore, residual confounding resulting from unmeasured area-level factors, such as detailed dietary pattern, environmental exposure, and genetic susceptibility, cannot be excluded. Lastly, our study focuses on the identification of risk areas and potentially associated geographical factors rather than causal inference. This ecological analysis may result in ecological fallacy when findings are interpreted as the association at the individual level. Thus, caution is needed to interpret our findings for the causal association and future studies based on individual data should investigate the association of individual- and area-level risk factors with GC development.

## 5. Conclusions

Gastric cancer is attributed to major health burden globally and can extensively benefit from its preventive nature. This study examined the locations of high- and low-risk areas of GC incidence and related geographic characteristics between the two time periods that are separated by increasing and decreasing GC incidence affected by the nationwide cancer prevention programs in South Korea. The expansion of low-risk areas and the changing profiles of associated lifestyle and environmental factors between periods are aligned with the potential population-level impact of nationwide prevention strategies. Findings highlight areas with persistent high risk, suggesting the need for locally tailored interventions targeting the socio-behavioral and environmental determinants.

## Supporting information

S1 Text Details of statistical methods to assess spatial autocorrelation and spatial clusters. (DOCX)

S2 Text Indirect age standardization of district-level gastric cancer screening participation. (DOCX)

S3 Text Calculation of standardized mean differences and confidence intervals. (DOCX)

S1 Table Previous studies of spatial clustering and regression for gastric cancer incidence. (DOCX)

S2 Table List of geographic characteristics in South Korea and their available years and data sources for 2009–2013 and 2014–2018. (DOCX)

S3 Table Approaches of neighbor definition based on spatial weights in spatial clustering analysis. (DOCX)

S4 Table Detailed geographic characteristics in high- and low-risk areas and their differences by 2009–2013 and 2014–2018. (DOCX)

S5 Table Geographic characteristics for 2009–2013 between high- and low-risk areas identified based on gastric cancer for 2014–2018. (DOCX)

S6 Table Associations of geographic characteristics and age-standardized gastric cancer incidence rates in 2009–2013 and 2014–2018, and their differences between two periods across 243 districts. (DOCX)

S1 Fig Global Moran’s I statistics against the number of neighboring districts. (DOCX)

S2 Fig Maps of clustered districts based on local Moran’s I (top) and Getis-Ord Gi (bottom) for age-standardized gastric cancer incidence rates in South Korea for 2009–2013 and 2014–2018. (DOCX)

S3 Fig Maps of high- and low-risk areas of age-standardized incidence rates for gastric cancer across 243 districts in South Korea for 2009–2013 and 2014–2018 by alternative spatial weight matrices. (DOCX)

S4 Fig Maps of high- and low-risk areas of age-standardized incidence rates for gastric cancer across 243 districts in South Korea for 2009–2013 and 2014–2018 with progressive application of permutation test and/or multiplicity adjustment. (DOCX)

S5 Fig Box plots of four geographic characteristics between two periods (2009–2013 and 2014–2018) in the districts whereby risk clusters changed (high to non-significant (NS), NS to low, low to NS, and NS to high-risk areas) for age-standardized gastric cancer incidence rates. (DOCX)

S6 Fig Map of the districts where clusters changed (high to non-significant (NS), NS to low, low to NS, and NS to high-risk areas) for age-standardized gastric cancer incidence rates. (DOCX)

S7 Fig Maps of gastric cancer risk clusters across 243 districts and three provinces with high *H. pylori* eradication in South Korea by 2009–2013 and 2014–2018. (DOCX)
