# Supplementary material for: Spatio-temporal changes in clusters of gastric cancer incidence: The impact of nationwide cancer control programs in South Korea
Source: PLoS One. 2026 Jun 16;21(6):e0349384. doi: 10.1371/journal.pone.0349384 (PMC13271449; doi:10.1371/journal.pone.0349384)
Supplement: S1 Text — (DOCX) [file pone.0349384.s001.docx]

**S1 Text.** Details of statistical methods to assess spatial autocorrelation and spatial clusters

- 1. **Global Moran’s I**

Global Moran’s I measures overall spatial autocorrelation based on both district locations and the attribute values of interest (e.g., gastric cancer incidence) simultaneously [1,2].

Global Moran’s I = $\frac{n\sum_{i=1}^{n} \sum_{j=1}^{n} w_{i,j}z_{i}z_{j}}{S_{0}*\sum z_{i}^{2}}$ (1)

$n$: total number of districts

$w_{i,j}$: spatial weight between district $i$ and $j$

$z_{i(j)}=x_{i(j)}-\bar{x}$ : deviation of an attribute for district $i$(or $j$) from the global mean

S_0_ = $\sum_{i=1}^{n} \sum_{j=1}^{n} w_{i,j}$ : aggregate of all spatial weights

The calculation of global Moran’s I involves measuring deviations from the mean for each district and computing weighted cross-products between neighboring districts (Equation 1). Suppose ‘i’ is a target district and ‘j’ is one of its neighboring districts. A positive cross-product occurs when both i and j have values above or below the mean, while a negative cross-product occurs when one is above and the other below. Greater deviations result in larger cross-products. These cross-products are then multiplied by the spatial weight (w_i,j_) between districts i and j and summed to produce the numerator, while the denominator serves as a normalization factor, incorporating both the sum of squared deviations and spatial weights to standardize the index between -1 and +1. A positive Moran’s I indicates clustering of similar values (e.g., high values near high values) and a negative Moran’s I suggests dispersion (e.g., high values near low values), while a value near zero indicates spatial randomness.

$z_{I}=\frac{I-E(I)}{\sqrt{V(I)}}$ (2)

$I$: observed global Moran’s I

$E\left( I \right)=-1/(n-1)$: expected global Moran’s I

$\sqrt{V(I)}=V\left( I \right)=E\left[ I^{2} \right]-E{[I]}^{2}$: standard deviation of global Moran’s I under randomness

After computing the global Moran’s I index, the tool calculates its expected value under a random spatial distribution and compares it to the observed index. Using the number of districts and the index variance, the z-score and p-value assess whether the observed spatial pattern is statistically significant (Equation 2). The null hypothesis assumes that the value of interest is randomly distributed across the study area.

If the z-score is positive and the p-value is statistically significant, it suggests that high or low values are more clustered than expected under a random spatial process. Conversely, if the z-score is negative and p-value is statistically significant, it suggests a dispersed spatial pattern where high values tend to be near low values, and vice versa.

- 1. **Local Moran’s I**

Local Moran’s I identifies localized clusters and spatial outliers by assessing spatial autocorrelation at each individual location by comparing a location’s deviation from the mean to the deviations of its neighbors [2-4].

Local Moran’s I (I_i_) = $\frac{x_{i}-\bar{x}}{s_{i}^{2}}\sum_{j=1, j\neq i}^{n} w_{i,j}(x_{j}-\bar{x})$ (3)

$n$: total number of districts

$z_{i}$=$x_{i(j)}-\bar{x}$ : deviation of an attribute for district $i$ from its global mean

$w_{i,j}$: spatial weight between district $i$ and $j$

$\sum_{j=1, j\neq i}^{n} w_{i,j}(x_{j}-\bar{x})$ : weighted sum of neighboring deviations

$s_{i}^{2}=\frac{\sum_{j=1, j\neq i}^{n} {(x_{j}-\bar{x})}^{2}}{n-1}$ : variance calculated by excluding location $i$ to prevent self-influence

The statistic ‘$I_{i}$’ is normalized using local variance to ensure comparability across different locations (Equation 3). The weighted sum of neighboring deviations accounts for spatial dependence, allowing for the identification of clusters and outliers. A positive ‘$I_{i}$’ indicates that a target district is surrounded by neighboring districts with similarly high or low attribute values, classifying it as a cluster. On the other hand, a negative ‘$I_{i}$’ value suggests that a district is surrounded by dissimilar values, identifying it as a spatial outlier.

$z_{I_{i}}=\frac{I_{i}-E(I_{i})}{\sqrt{V(I_{i})}}$ (4)

$I_{i}$: observed local Moran’s I

$E\left( I_{i} \right)=-\frac{\sum_{j=1, j\neq i}^{n} w_{i,j}}{n-1}$: expected local Moran’s I

$\sqrt{V(I_{i})}=V\left( I_{i} \right)=E\left[ I_{i}^{2} \right]-E{[I_{i}]}^{2}$: standard deviation of local Moran’s I under randomness

After calculating the local Moran’s I index, the tool computes its expected value under a random spatial distribution and compares it to the observed index for each location (Equation 4). Using the number of districts and the local index variance, the z-score and p-value assess whether the observed spatial pattern at each location is statistically significant. The null hypothesis assumes that the value of interest is randomly distributed across the study area.

If the z-score is positive and the p-value is statistically significant, a local cluster exists, where a location is surrounded by neighbors with similar values (high-high or low-low). Conversely, if the z-score is negative and the p-value is statistically significant, it is defined as a spatial outlier, where a location has dissimilar neighboring values (high-low or vice versa).

- 1. **Getis-Ord-Gi***

The Getis-Ord Gi∗ approach evaluates the local sum of weighted values of interest in a target district ‘i’ and its neighbors ‘j’, and comparing it proportionally to the sum of overall districts [2,5,6].

Getis Ord Gi* (Gi*) = $\frac{\sum_{j=1}^{n} w_{i,j}x_{j}-\bar{x}\sum_{j=1}^{n} w_{i,j}}{s\sqrt{\frac{[\sum_{j=1}^{n} w_{i,j}^{2}-{(\sum_{j=1}^{n} w_{i,j})}^{2}]}{n-1}}}$ (5)

$n$: total number of districts

$w_{i,j}$: spatial weight between district $i$ and $j$

$x_{j}$: attribute value for district$j$

$\bar{x}$: global mean of an attribute

s =$\sqrt{\frac{\sum_{j=1}^{n} x_{j}^{2}}{n}-{(\bar{x)}}^{2}}$: standard deviation of an attribute

The numerator computes the difference between the spatially weighted sum of neighboring values and the expected value under a random spatial distribution (Equation 5). The denominator normalizes this difference by accounting for the standard deviation and the variation of spatial weight. The Getis-Ord-Gi* statistic itself is a z-score, and a two-tailed test at a 95% confidence level is commonly used. If the p-value is statistically significant and the z-score is positive, high values exhibit significant spatial clustering. Conversely, a negative z-score indicates significant clustering of low values.

**References**

1. Esri. How Spatial Autocorrelation (Global Moran's I) works [cited 2025 March 15]. Available from: <https://pro.arcgis.com/en/pro-app/latest/tool-reference/spatial-statistics/h-how-spatial-autocorrelation-moran-s-i-spatial-st.htm>.

2. Mitchell A, Griffin LS. The ESRI Guide to GIS analysis, Volume 2: Spatial measurements and statistics. ESRI Press. 2021;2.

3. Esri. How Cluster and Outlier Analysis (Anselin Local Moran's I) works [cited 2025 March 15]. Available from: <https://pro.arcgis.com/en/pro-app/latest/tool-reference/spatial-statistics/h-how-cluster-and-outlier-analysis-anselin-local-m.htm>.

4. Anselin L. Local indicators of spatial association—LISA. Geogr Anal. 1995;27(2):93-115.

5. Ord JK, Getis A. Local spatial autocorrelation statistics: distributional issues and an application. Geogr Anal. 1995;27(4):286-306.

6. Esri. How Hot Spot Analysis (Getis-Ord Gi*) works [cited 2025 March 15]. Available from: <https://pro.arcgis.com/en/pro-app/latest/tool-reference/spatial-statistics/h-how-hot-spot-analysis-getis-ord-gi-spatial-stati.htm>.
