# Supplementary material for: Spatio-temporal changes in clusters of gastric cancer incidence: The impact of nationwide cancer control programs in South Korea
Source: PLoS One. 2026 Jun 16;21(6):e0349384. doi: 10.1371/journal.pone.0349384 (PMC13271449; doi:10.1371/journal.pone.0349384)
Supplement: S2 Text — (DOCX) [file pone.0349384.s002.docx]

**S2 Text.** Indirect age standardization of district-level gastric cancer screening participation

We derived district-level gastric cancer–specific screening participation rate, defined as the proportion of individuals aged ≥ 40 years who underwent gastric cancer screening among the eligible population in the corresponding year. Because district-level age-specific gastric cancer screening counts were unavailable, direct age standardization was not feasible. We therefore applied indirect age standardization using national age-specific gastric cancer screening rates as the reference, together with mid-year district populations stratified into 5-year age groups, to account for demographic differences across districts.

For each district, the expected number of screened individuals ($E_{j}$)was calculated as:

$$E_{j}=\sum_{i} (n_{ij}\times r_{i})$$

where $n_{ij}$ denotes the population of district $j$ in age group $i$, and $r_{i}$ represents the corresponding national age-specific gastric cancer screening rate. The indirectly age-standardized screening rate (ISR) was then calculated as:

$$ISR_{j}=(\frac{O_{j}}{E_{j}})\times R$$

where $O_{j}$is the observed number of screened individuals in district $j$ and $R$ is the national crude screening rate.
