# Supplementary material for: Spatio-temporal changes in clusters of gastric cancer incidence: The impact of nationwide cancer control programs in South Korea
Source: PLoS One. 2026 Jun 16;21(6):e0349384. doi: 10.1371/journal.pone.0349384 (PMC13271449; doi:10.1371/journal.pone.0349384)
Supplement: S3 Text — (DOCX) [file pone.0349384.s003.docx]

**S3 Text.** Calculation of standardized mean differences and confidence intervals

Standardized mean difference (SMD) of GC incidence (X) across n districts between high- and low-risk areas (H and L, respectively) was calculated as follows:

$$\text{SMD}=\frac{X_{H}-X_{L}}{S_{\text{pooled}}},$$

$$S_{\text{pooled}}=\sqrt{\frac{(n_{H}-1)s_{H}^{2}+(n_{L}-1)s_{L}^{2}}{n_{H}+n_{L}-2}}$$

The variance of SMD was estimated as:

$$\mathrm{Var}(\text{SMD})=\frac{n_{H}+n_{L}}{n_{H}n_{L}}+\frac{\text{SMD}^{2}}{2(n_{H}+n_{L}-2)}$$

and 95% confidence intervals were computed as:

$\text{SMD}\pm1.96\times\sqrt{\mathrm{Var}(\text{SMD})}$
