# Supplementary material for: Spatio-temporal changes in clusters of gastric cancer incidence: The impact of nationwide cancer control programs in South Korea
Source: PLoS One. 2026 Jun 16;21(6):e0349384. doi: 10.1371/journal.pone.0349384 (PMC13271449; doi:10.1371/journal.pone.0349384)
Supplement: S1 Table — (DOCX) [file pone.0349384.s004.docx]

**S1 Table.** Previous studies of spatial clustering and regression for gastric cancer incidence

| **Spatial analysis** | **Author (year)** | **Study area** | **Study period** | **Analysis method** | **Spatial unit** | **Major findings** | **Geographical variables** | | | | | | |  |
| --- | --- | --- | --- | --- | --- | --- | --- | --- | --- | --- | --- | --- | --- | --- |
|  |  |  |  |  |  |  | **D** | **S** | **L** | **M** | **HI** | **HS** | **P** | |
| Clustering | Han  (2023) [1] | China | 2016 | Local Moran’s I | Province | High-risk clusters in the east–west belt. |  |  |  |  |  |  |  | |
|  | Faramarzi  (2024) [2] | Iran | 2014-2017 | SSS | County | High-risk areas in northern and northwestern regions. |  |  |  |  |  |  |  | |
|  | Solimany  (2018) [3] | Iran | 2009-2014 | SSS | County | High-risk areas in the east, and low-risk areas in the west. |  |  |  |  |  |  |  | |
|  | Moradzadeh  (2022) [4] | Iran | 2009-2014 | SSS | Census block | High-risk areas in the central and southwest. |  |  |  |  |  |  |  | |
|  | Soleimani  (2023) [5] | Iran | 2015-2017 | Getis-Ord-GI* | County | Changed hotspots from 2015 to 2017 |  |  |  |  |  |  |  | |
|  | Dadashi  (2023) [6] | Iran | 2017-2018 | Getis-Ord-GI* | County | A low-risk cluster in women. |  |  |  |  |  |  |  | |
| Regression | Fakanye  (2021) [7] | Canada | 1992-2016 | INLA and ICAR | Regional health authority district (RHAD) | High risk areas identified after adjusting for socioeconomic score index and indigenous and immigrant population. Steady overall risk estimates across provinces over time but apparent changes at the RHAD level | O | O |  |  |  |  |  | |
|  | Ko  (2016) [8] | South Korea  (Busan city) | 2004-2008 | CAR | District and neighborhood | Similar relative risk with adjacent districts after controlling for age, sex, year, and deprivation index | O | O |  |  |  |  |  | |
| Clustering and exploration of responsible geographic characteristics | Lee  (2019) [9] | Korea | 2009-2013 | SLM and SEM | District | High-risk clusters identified in non-urban areas from a southern region mostly with older adults and low-risk clusters mostly located in the Seoul metropolitan area.  Proportion of manufacturing workers and buildings constructed before 1995, and the number of people per one medical personnel associated with GC incidence. | O |  |  |  | O |  | O | |
|  | Song  (2023) [10] | South Korea | 1999-2013 | SSS | District | High-risk clusters in the south-central region for 1999-2013, and reduction of cluster size after excluding the influence of geographic characteristics. | O | O | O |  |  |  | O | |
|  | Nguyen (2023) [11] | South Korea | 1999-2013 | SSS | District | High-risk clusters in the central region and retained for 1999-2013, showing more older adults, unemployment rate, and GC screening, but less current smokers and hospital beds. | O | O | O |  | O | O |  | |
|  | Huang (2023a) [12] | China  (Gansu province) | 2013-2021 | SSS and GWR | County | High-and low-risk areas in northern and southern areas, respectively.  Rainfall and ambient temperature, healthcare resource, Gross Domestic Product, and primary industry output correlated with GC incidence. |  | O |  |  | O |  | O | |
|  | Huang  (2023b) [13] | China  (Gansu province) | 2013-2021 | SSS and LISA | City | Hotspot clusters identified in the north and southwest, and cold spot clusters in the south.  Similar cluster locations using different approaches  Medical resource allocation and socioeconomic status identified as key determinants |  | O |  |  | O |  | O | |
|  | Dominguez  (2019) [14] | Honduras | 2002-2013 | Getis-Ord-Gi* and SSS | Village | High-risk clusters in the west aligned with the clustering regions of CagA-positive *H. pylori* infections. |  |  |  | O |  |  |  | |

D: Demography; S: Socioeconomic status; L: Lifestyle; M: Medical status; HI: Healthcare infrastructure; HS: Health screening; P: Physical environment; GC: Gastric cancer. SSS: spatial scan statistics. INLA: integrated nested Laplace approximation. ICAR: intrinsic conditional autoregressive. CAR: conditional autoregressive. SLM: spatial lag model. SEM: spatial error model. GWR: geographically weighted regression. Lisa: local indicator of spatial association.

**References**

1. Han W, Yang C, Chen W, Wu P, Xue F, Wang Z, et al. Spatial co-occurrence of multiple cancers: a population-based study using national cancer registry data in China. Res Sq. 2023:1-12.

2. Faramarzi S, Kiani B, Faramarzi S, Firouraghi N. Cancer patterns in Iran: a gender-specific spatial modelling of cancer incidence during 2014–2017. BMC Cancer. 2024;24(1):191.

3. Solimany A, Khoramdad M, Khademi N, Delpisheh A. Spatio-temporal study of gastric cancer incidence in Kermanshah Province, Iran during the years 2009-2014. Asian Pac J Cancer Prev. 2018;19(10):2871.

4. Moradzadeh R, Shamsi M, Heidari S. Spatial cluster analysis of stomach neoplasms in the center of iran based on a population-based study, 2009-2014: Application of the poisson-based probability model. Int J Prev Med. 2022;13(1):28.

5. Soleimani M, Saeini MR, Jalilvand A. Spatial and temporal analysis of gastric cancer incidence in northwest Iran. GeoJournal. 2023;88:4555–72.

6. Dadashi A, Mohammadi A, MohammadEbrahimi S, Bergquist R, Shamsoddini A, Hesami A, et al. ‎Spatial analysis of the 10 most prevalent cancers in north-eastern Iran, 2017–2018‎. J Spat Sci. 2023;68(2):281-301.

7. Fakanye O, Singh H, Desautels D, Torabi M. Geographical variation and factors associated with gastric cancer in Manitoba. PLoS One. 2021;16(7):e0253650.

8. Ko YG. Cancer incidence and mortality estimations in Busan by using Spatial multi-level model: Department of Statistics, Pukyong National University; 2016.

9. Lee SH, Kim K. Analysis of association between cancer incidence and geographical and environmental characteristics using spatial statistical techniques. J Korean Categor Assoc. 2019;19(3):57-74.

10. Song I, Yoo EH, Jung I, Oh JK, Kim SY. Role of geographic characteristics in the spatial cluster detection of cancer: Evidence in South Korea, 1999–2013. Environ Res. 2023;236:116841.

11. Nguyen CT, Song I, Jung I, Choi YJ, Kim SY. Changes in spatial clusters of cancer incidence and mortality over 15 years in South Korea: Implication to cancer control. Cancer Med. 2023;12(16):17418-27.

12. Huang B, Ding F, Liu J, Li Y. Government drivers of gastric cancer prevention: The identification of risk areas and macro factors in Gansu, China. Prev Med Rep. 2023a;36:102450.

13. Huang B, Liu J, Ding F, Li Y. Epidemiology, risk areas and macro determinants of gastric cancer: a study based on geospatial analysis. Int J Health Geogr. 2023b;22(1):32.

14. Dominguez RL, Cherry CB, Estevez-Ordonez D, Mera R, Escamilla V, Pawlita M, et al. Geospatial analyses identify regional hot spots of diffuse gastric cancer in rural Central America. BMC Cancer. 2019;19(545).
