# Supplementary material for: Spatio-temporal changes in clusters of gastric cancer incidence: The impact of nationwide cancer control programs in South Korea
Source: PLoS One. 2026 Jun 16;21(6):e0349384. doi: 10.1371/journal.pone.0349384 (PMC13271449; doi:10.1371/journal.pone.0349384)
Supplement: S2 Table — (DOCX) [file pone.0349384.s005.docx]

**S2 Table.** List of geographic characteristics in South Korea and their available years and data sources for 2009–2013 and 2014–2018

| **District-level Characteristics** | **Year** | | **Data sources** |
| --- | --- | --- | --- |
|  | **2009-2013** | **2014-2018** |  |
| Demography |  |  |  |
| % of older adults ≥ 65 years | 2010 | 2015 | KOSIS |
| Sex ratio | 2010 | 2015 | KOSIS |
| Population density^a^ | 2010 | 2015 | KOSIS |
| % of urban-dwelling population | 2010 | 2015 | KCHS |
| Socioeconomic status |  |  |  |
| Growth regional domestic product per capita (1000 USD/person) | 2010 | 2015 | KOSIS |
| % of higher education^b^ | 2010 | 2015 | KOSIS |
| Lifestyle |  |  |  |
| % of breakfast ≥5 times/week^c^ | 2010 | 2015 | KCHS |
| % of low-salt preference^c,d^ | 2010 | 2015 | KCHS |
| % of current smokers^c,e^ | 2010 | 2015 | KCHS |
| % of heavy drinking^c,f^ | 2010 | 2015 | KCHS |
| % of moderate to vigorous physical activity^c,g^ | 2010 | 2015 | KCHS |
| % of regular walking^c,h^ | 2010 | 2015 | KCHS |
| % of self-reported obesity^c,i^ | 2010 | 2015 | KCHS |
| Medical status |  |  |  |
| % of doctor’s diagnosis of hypertension^c^ | 2010 | 2015 | KCHS |
| % of doctor’s diagnosis of diabetes^c^ | 2010 | 2015 | KCHS |
| % of doctor’s diagnosis of dyslipidemia^c^ | 2011 | 2015 | KCHS |
| Healthcare infrastructure |  |  |  |
| Number of hospital beds per 1000 people | 2010 | 2015 | KOSIS |
| Number of medical personnel per 1000 people | 2010 | 2015 | KOSIS |
| Medical accessibility |  |  |  |
| % of unmet healthcare needs^c,j^ | 2011 | 2015 | KCHS |
| Health screening |  |  |  |
| % of cancer screening examinees for the previous 2 years^c^ | 2010 | 2015 | KCHS |
| % of health screening examinees for the previous 2 years^c^ | 2010 | 2015 | KCHS |
| % of gastric cancer screening examinees^k^ | 2010 | 2015 | KOSIS |
| Physical environment |  |  |  |
| % of urban forest coverage within residential area^i^ | 2011 | 2015 | KCHS |

KOSIS: Korean Statistical Information Service. KCHS: Korea Community Health Survey.

^a^(Number of population/land area [km^2^]); ^b^≥15-year-old who have completed at least a college degree; ^c^All values are standardized by age based on the population projections in 2005; ^d^Self-reported low-salt preference by 1) taking low levels of salt intake, 2) not adding salt or soy sauce to the dishes served on tables; and 3) not dipping to soy sauce for fried foods; ^e^Individuals who had smoked at least 100 cigarettes (five packs) during their lifetime and who reported current smoking at the time of the survey; ^f^Individuals who reported alcohol consumption within the past year and drank alcohol more than twice per week, consuming more than seven standard drinks (or five cans of beer) per occasion for men and more than five standard drinks (or three cans of beer) per occasion for women; ^g^Vigorous physical activity ≥3 times/week and ≥20 minutes/day, or moderate physical activity ≥5 times/week and ≥30 minutes/day; ^h^Walking ≥5 times/week and ≥30 minutes/day; ^i^Self-reported body mass index ≥25 kg/m^2^; ^j^Individuals who believed they required medical care (excluding dental care) but were unable to receive it within the past year; ^i^ The area of urban forests located within living areas that are easily accessible to residents with minimal time and cost burden, calculated as: (area of urban forest within residential areas / total administrative area) × 100; ^k^Individuals aged ≥40 years who underwent gastric cancer screening among the eligible population in the corresponding year. Indirect age-standardization was applied using the mid-year population of the corresponding year, grouped into 5-year age categories **(S2 Text)**.
