# Supplementary material for: Spatio-temporal changes in clusters of gastric cancer incidence: The impact of nationwide cancer control programs in South Korea
Source: PLoS One. 2026 Jun 16;21(6):e0349384. doi: 10.1371/journal.pone.0349384 (PMC13271449; doi:10.1371/journal.pone.0349384)
Supplement: S3 Table — (DOCX) [file pone.0349384.s006.docx]

**S3 Table.** Approaches of neighbor definition based on spatial weights in spatial clustering analysis

| **Category** | **Method** | **Identification of neighboring districts** |
| --- | --- | --- |
| Adjacency-based | Queen contiguity | The districts that share either edges or corners with a target district [1-3]. |
|  | Rook contiguity | The districts that share edges only as a restrictive approach compared to Queen contiguity [4]. |
| Distance-based | k-nearest neighbors | The k closest districts [5]. It ensures that each district has exactly k neighbors, thereby avoiding the isolation that may occur under contiguity-based schemes [6].  An optimal number of k is determined based on the maximum spatial autocorrelation [7] or the average number of contiguous neighbors as a practical guidance [8]. |
|  | Distance bands | The districts within a specified threshold distance [9]. |

**References**

1. Yang E, Bae H, Ryu D. What makes the level of particulate matter emissions worse in Korea? Rom J Econ Forecast. 2022;25(3):128-43.

2. Ju S, Noh J, Kim C, Heo J. Local spatial autocorrelation analysis of 3 disease prevalence: a case study of Korea. J Health Info Stat. 2017;42(4):301-8.

3. Tsai PJ, Lin M-L, Chu CM, Perng CH. Spatial autocorrelation analysis of health care hotspots in Taiwan in 2006. BMC Public Health. 2009;9:464.

4. Kang JE, Yoon D, Bae H-J. Evaluating the effect of compact urban form on air quality in Korea. Environ Plann B Urban Anal City Sci. 2019;46(1):179-200.

5. Ertur C, Le Gallo J. An exploratory spatial data analysis of European regional disparities, 1980–1995: Springer; 2003. 55-97 p.

6. Bao Y, Huang I, Li Q, Zhang Z, Xing Y, Hou D, et al. A framework for modeling county-level COVID-19 transmission. Front Public Health. 2025;13:1608360.

7. Kim J, Zhang M. Determining transit’s impact on Seoul commercial land values: An application of spatial econometrics. Int Real Estate Rev. 2005;8(1):1-26.

8. Wan Y, Tan X, Shu H. Finding and evaluating community structures in spatial networks. ISPRS Int J Geoinf. 2023;12(5):187.

9. Park EH, Kang MJ, Jung KW, Yun EH, Kim HJ, Kong HJ, et al. Regional disparities in major cancer incidence in South Korea, 1999–2018. Epidemiol Health. 2023;45:e2023089.
