# Supplementary material for: Spatio-temporal changes in clusters of gastric cancer incidence: The impact of nationwide cancer control programs in South Korea
Source: PLoS One. 2026 Jun 16;21(6):e0349384. doi: 10.1371/journal.pone.0349384 (PMC13271449; doi:10.1371/journal.pone.0349384)
Supplement: S4 Table — (DOCX) [file pone.0349384.s007.docx]

**S4 Table.** Detailed geographic characteristics in high- and low-risk areas and their differences by 2009–2013 and 2014–2018

| **District-level characteristics** | **2009–2013** | | | | | **2014–2018** | | | | | **HL & PP difference^h^** | |
| --- | --- | --- | --- | --- | --- | --- | --- | --- | --- | --- | --- | --- |
|  | **High-risk area**  **(N=31)^f^** | **Low-risk area (26)** | **HL difference^g^** | | | **High-risk area (28)** | **Low-risk area**  **(37)** | **HL difference^g^** | | |  |  |
|  | **Mean (SD)** | **Mean (SD)** | **AAD^a^** | **ARD^b^** | **SMD (95% CI)^c^** | **Mean (SD)** | **Mean (SD)** | **AAD^a^** | **ARD^b^** | **SMD (95% CI)^c^** | **AAD^a^** | **ARD^b^** |
| Gastric cancer incidence rate |  |  |  |  |  |  |  |  |  |  |  |  |
| Age-standardized incidence rate per 100,000 | 95.9 (6.0) | 73.4 (6.9) | 22.5^d^ | 30.7 | 3.5 (2.7, 4.4) | 79.2 (7.0) | 61.1 (3.1) | 18.1^d^ | 29.6 | 3.5 (2.7, 4.3) | –4.4 | –1.0 |
| Demography |  |  |  |  |  |  |  |  |  |  |  |  |
| % of older adults ≥ 65 years | 17.5 (7.3) | 12.1 (4.0) | 5.4 | 44.6 | 0.9 (0.3, 1.4) | 22.3 (7.5) | 12.5 (2.6) | 9.8^d^ | 78.4 | 1.9 (1.3, 2.4) | 4.4^e^ | 33.8 |
| Sex ratio | 101.0 (3.6) | 101.7 (4.6) | –0.7 | –0.7 | –0.2 (–0.7, 0.3) | 99.3 (2.9) | 98.3 (3.9) | 1.0 | 1.0 | 0.3 (–0.2, 0.8) | 1.7^e^ | 1.7 |
| Population density | 7.5 (12.8) | 68.4(76.3) | –60.9 | –89.0 | –1.2 (–1.7, –0.6) | 5.6 (12.1) | 105.0 (75.8) | –99.4^d^ | –94.7 | –1.7 (–2.3, –1.1) | –38.5 | –5.6 |
| % of urban-dwelling population | 64.7 (25.5) | 84.8 (20.8) | –20.1 | –23.7 | –0.9 (–1.4, –0.3) | 57.7 (24.6) | 95.9 (11.5) | –38.2^d^ | –39.8 | –2.1 (–2.7, –1.5) | –18.1 | –16.1 |
| Socio-economic status |  |  |  |  |  |  |  |  |  |  |  |  |
| Growth regional domestic product per capita (1,000 USD/person) | 17.7 (7.2) | 27.1 (46.8) | –9.4 | –34.7 | –0.3 (–0.8, 0.2) | 21.5 (9.2) | 31.4 (49.2) | –9.9 | –31.5 | –0.3 (–0.8, 0.2) | –0.5 | 3.2 |
| % of higher education | 21.3 (8.9) | 30.8 (9.7) | –9.5^d^ | –30.8 | –1.0 (–1.6, –0.5) | 24.6 (8.5) | 42.4 (10.2) | –17.8^d^ | –42.0 | –1.9 (–2.5, –1.3) | –8.3 | –11.1 |
| Lifestyle |  |  |  |  |  |  |  |  |  |  |  |  |
| % of breakfast ≥5 times/week | 73.5 (4.7) | 71.3 (3.9) | 2.2 | 3.1 | 0.5 (0, 1.0) | 69.5 (5.8) | 63.5 (3.9) | 6.0^d^ | 9.4 | 1.3 (0.7, 1.8) | 3.8 | 6.4 |
| % of low-salt preference | 9.7 (2.4) | 9.4 (2.1) | 0.3 | 3.2 | 0.1 (–0.4, 0.7) | 10.8 (2.7) | 12.3 (1.9) | –1.5 | –12.2 | –0.6 (–1.1, –0.1) | –1.8 | –15.4 |
| % of current smoking | 25.9 (2.5) | 25.2 (2.3) | 0.7 | 2.8 | 0.3 (–0.2, 0.8) | 21.7 (2.6) | 21.1 (3.0) | 0.6 | 2.8 | 0.2 (–0.3, 0.7) | –0.1 | 0.1 |
| % of heavy drinking | 14.1 (3.6) | 15.6 (3.2) | –1.5^d^ | –9.6 | –0.4 (–1.0, 0.1) | 19.3 (3.5) | 17.9 (2.7) | 1.4 | 7.8 | 0.5 (–0.05, 0.9) | 2.9^e^ | 17.4 |
| % of moderate to vigorous physical activity | 22.4 (4.6) | 23.0 (8.2) | –0.6 | –2.6 | –0.1 (–0.6, 0.4) | 24.2 (6.9) | 21.8 (3.4) | 2.4 | 11.0 | 0.5 (–0.03, 1.0) | 3.0 | 13.6 |
| % of regular walking | 39.2 (10.7) | 48.7 (11.8) | –9.5 | –19.5 | –0.8 (–1.4, –0.3) | 39.7 (10.2) | 50.0 (10.2) | –10.3^d^ | –20.6 | –1.0 (–1.5, –0.5) | –0.8 | –1.1 |
| % of self-reported obesity | 22.1 (3.2) | 24.0 (3.1) | –1.9 | –7.9 | –0.6 (–1.1, –0.1) | 26.2(2.8) | 24.9 (1.9) | 1.3^d^ | 5.2 | 0.5 (0.05, 1.0) | 3.2 | 13.1 |
| Medical status |  |  |  |  |  |  |  |  |  |  |  |  |
| % of doctor’s diagnosis of hypertension | 14.3 (2.0) | 15.6 (1.2) | –1.3 | –8.3 | –0.7 (–1.3, –0.2) | 15.6 (2.0) | 15.2 (1.5) | 0.4 | 2.6 | 0.2 (–0.3, 0.7) | 1.7^e^ | 11.0 |
| % of doctor’s diagnosis of diabetes | 5.5 (1.0) | 5.9 (0.8) | –0.4 | –6.8 | –0.5 (–1.0, 0.02) | 6.1 (0.7) | 5.8 (1.0) | 0.3 | 5.2 | 0.3 (–0.2, 0.8) | 0.7 | 12.0 |
| % of doctor’s diagnosis of dyslipidemia | 7.3 (1.9) | 9.2 (1.9) | –1.9^d^ | –20.7 | –1.0 (–1.5, –0.4) | 10.1 (2.5) | 11.4 (1.4) | –1.3 | –11.4 | –0.7 (–1.2, –0.2) | 0.6 | 9.2 |
| Healthcare infrastructure |  |  |  |  |  |  |  |  |  |  |  |  |
| Number of hospital beds per 1000 people | 12.5 (7.7) | 8.6 (3.8) | 3.9 | 45.3 | 0.6 (0.1, 1.2) | 14.6 (6.5) | 9.5 (4.2) | 5.1 | 53.7 | 1.0 (0.4, 1.5) | 1.2 | 8.3 |
| Number of medical personnel per 1000 people | 2.0 (0.8) | 2.8 (3.0) | –0.8 | –28.6 | –0.4 (–0.9, 0.2) | 2.2 (0.8) | 3.6 (3.3) | –1.4 | –38.9 | –0.5 (–1.0, –0.03) | –0.6 | –10.3 |
| Medical accessibility |  |  |  |  |  |  |  |  |  |  |  |  |
| % of unmet healthcare needs | 12.3 (4.7) | 14.0 (3.2) | –1.7 | –12.1 | –0.4 (–1.0, 0.1) | 12.6 (4.5) | 11.3 (2.5) | 1.3 | 11.5 | 0.4 (–0.1, 0.9) | 3.0 | 23.6 |
| Health screening |  |  |  |  |  |  |  |  |  |  |  |  |
| % of cancer screening examinees for the previous 2 years | 44.7 (3.3) | 44.0 (2.7) | 0.7 | 1.6 | 0.2 (–0.3, 0.8) | 50.0 (5.9) | 50.4 (5.0) | –0.4 | –0.8 | –0.1 (–0.6, 0.4) | –1.1 | –2.4 |
| % of gastric cancer screening examinees | 50.0 (3.4) | 44.4 (3.5) | 5.6^d^ | 12.6 | 1.6 (1.0, 2.2) | 61.3 (3.6) | 54.6 (2.9) | 6.7^d^ | 12.3 | 2.1 (1.5, 2.7) | 1.1 | –0.3 |
| % of health screening examinees for the previous 2 years | 55.3 (4.6) | 57.3 (4.0) | –2.0 | –3.5 | –0.5 (–1.0, 0.1) | 61.3 (5.8) | 63.3 (4.1) | –2.0 | –3.2 | –0.4 (–0.9, 0.1) | 0 | 0.3 |
| Physical environments |  |  |  |  |  |  |  |  |  |  |  |  |
| % of urban forest coverage within residential area | 1.5 (1.6) | 2.9 (3.3) | –1.4 | –48.3 | –0.6 (–1.1, –0.1) | 1.7 (1.8) | 6.9 (11.5) | –5.2^d^ | –75.4 | –0.6 (–1.1, –0.1) | –3.8 | –27.1 |

^a^Average absolute difference in characteristics between high- and low-risk areas calculated as (average of high − low) for each period.

^b^Average relative difference in characteristics between high- and low-risk areas calculated as (average of high − low)*100 / low for each period.

^c^Standardized mean difference (SMD) as the difference in means between high- and low-risk areas divided by pooled standard deviation; SMD ≥ 0.2, 0.5, and 0.8 indicating a small, median, and large difference, respectively (Cohen 1988 [1]); 95% CIs indicating 95% confidence intervals.

^d^Statistical significance assessed by comparing high- and low-risk areas within each period using Student’s t-test (all variables in lifestyle, medical status, medical accessibility, and health screening categories and the number of hospital beds in healthcare infrastructure) or the Mann–Whitney U test (all variables in demography, socioeconomic status, and physical environment categories and the number of medical personnels in healthcare infrastructure), with a Bonferroni-corrected significance threshold of p < 0.00217 (0.05/23).

^e^Statistical significance assessed by an interaction by the status of clusters and periods across 36 districts identified as high- or low-risk areas in both periods

^f^Number of districts

^g^Difference between low- and high-risk areas

^h^Difference between low- and high-risk areas and between the two periods

**References**

1. Cohen J. Statistical power analysis for the behavioral sciences. 2nd ed. Mahwah, NJ, USA: Lawrence Erlbaum Associates; 2013.
