# Supplementary material for: Spatio-temporal changes in clusters of gastric cancer incidence: The impact of nationwide cancer control programs in South Korea
Source: PLoS One. 2026 Jun 16;21(6):e0349384. doi: 10.1371/journal.pone.0349384 (PMC13271449; doi:10.1371/journal.pone.0349384)
Supplement: S5 Table — (DOCX) [file pone.0349384.s008.docx]

**S5 Table.** Geographic characteristics for 2009–2013 between high- and low-risk areas identified based on gastric cancer for 2014–2018

| **District-level characteristics in 2010–2011** | **High-risk area**  **(N=28)^e^** | **Low-risk area (37)** | **HL Difference** | | |
| --- | --- | --- | --- | --- | --- |
|  | **Mean (SD)** | **Mean (SD)** | **AAD^a^** | **ARD^b^** | **SMD (95% CI)^c^** |
| Gastric cancer incidence rate |  |  |  |  |  |
| Age-standardized incidence rate per 100,000 | 93.7 (8.6)^d^ | 74.9 (4.4) | 18.8^d^ | 25.1 | 2.9 (2.2, 3.6) |
| Demography |  |  |  |  |  |
| % of older adults ≥ 65 years | 19.7 (7.2)^d^ | 10.0 (2.3) | 9.7^d^ | 97.0 | 1.9 (1.3, 2.5) |
| Sex ratio | 99.6 (3.0) | 99.5 (3.7) | 0.1 | 0.1 | 0.01 (-0.5, 0.5) |
| Population density | 5.7 (12.4)^d^ | 106.7 (78.9) | -101.0^d^ | -94.7 | -1.7 (-2.2, -1.1) |
| % of urban-dwelling population | 56.5 (24.5)^d^ | 95.3 (12.0) | -38.8^d^ | -40.7 | -2.1 (-2.7, -1.5) |
| Socio-economic status |  |  |  |  |  |
| Growth regional domestic product per capita (1,000 USD/person) | 16.7 (10.3) | 24.0 (40.2) | -7.3 | -30.4 | -0.2 (-0.7, 0.3) |
| % of higher education | 19.8 (9.1)^d^ | 37.8 (10.3) | -18.0^d^ | -47.6 | -1.8 (-2.4, -1.3) |
| Lifestyle |  |  |  |  |  |
| % of breakfast ≥5 times/week | 74.2 (5.0)^d^ | 70.1 (3.6) | 4.1^d^ | 5.8 | 1.0 (0.4, 1.5) |
| % of low-salt preference | 9.8 (2.6) | 10.1 (2.2) | -0.3 | -3.0 | -0.1 (-0.6, 0.4) |
| % of current smoking | 25.5 (2.3) | 24.1 (3.0) | 1.4 | 5.8 | 0.5 (0.02, 1.0) |
| % of heavy drinking | 13.4 (4.3) | 14.9 (3.4) | -1.5 | -10.1 | -0.4 (-0.9, 0.1) |
| % of moderate to vigorous physical activity | 24.6 (6.2)^d^ | 19.9 (4.6) | 4.7^d^ | 23.6 | 0.9 (0.4, 1.4) |
| % of regular walking | 41.0 (11.3) | 49.6 (11.4) | -8.6 | -17.3 | -0.8 (-1.3, -0.2) |
| % of self-reported obesity | 21.9 (2.3) | 22.4 (2.6) | -0.5 | -2.2 | -0.2 (-0.7, 0.3) |
| Medical status |  |  |  |  |  |
| % of doctor’s diagnosis of hypertension | 14.2 (1.6) | 15.4 (1.5) | -1.2 | -7.8 | -0.7 (-1.2, -0.2) |
| % of doctor’s diagnosis of diabetes | 5.2 (0.8) | 5.9 (0.8) | -0.7 | -11.9 | -0.7 (-1.2, -0.2) |
| % of doctor’s diagnosis of dyslipidemia | 7.5 (2.1)^d^ | 9.9 (1.5) | -2.4^d^ | -24.2 | -1.3 (-1.8, -0.7) |
| Healthcare infrastructure |  |  |  |  |  |
| Number of hospital beds per 1000 people | 11.8 (6.0) | 8.2 (3.6) | 3.6 | 43.9 | 0.7 (0.2, 1.3) |
| Number of medical personnel per 1000 people | 2.0 (0.7) | 3.2 (2.8) | -1.2 | -37.5 | -0.6 (-1.1, -0.1) |
| Medical accessibility |  |  |  |  |  |
| % of unmet healthcare needs | 13.3 (4.7) | 14.0 (3.2) | -0.7 | -5.0 | -0.2 (-0.7, 0.3) |
| Health screening |  |  |  |  |  |
| % of cancer screening examinees for the previous 2 years | 43.3 (4.3) | 44.1 (3.2) | -0.8 | -1.8 | -0.2 (-0.7, 0.3) |
| % of gastric cancer screening examinees | 49.3 (4.7)^d^ | 43.2 (3.1) | 6.1^d^ | 14.1 | 1.6 (1.0, 2.1) |
| % of health screening examinees for the previous 2 years | 54.3 (5.3) | 58.0 (4.3) | -3.7 | -6.4 | -0.8 (-1.3, -0.3) |
| Physical environments |  |  |  |  |  |
| % of urban forest coverage within residential area | 1.4 (1.7)^d^ | 4.8 (4.5) | -3.4^d^ | -70.8 | -0.9 (-1.5, -0.4) |

^a^Average absolute difference in characteristics between high- and low-risk areas calculated as (average of high − low) for each period.

^b^Average relative difference in characteristics between high- and low-risk areas calculated as (average of high − low)*100 / low for each period.

^c^Standardized mean difference (SMD) as the difference in means between high- and low-risk areas divided by pooled standard deviation; SMD ≥ 0.2, 0.5, and 0.8 indicating a small, median, and large difference, respectively (Cohen 1988 [1]); 95% CIs indicating 95% confidence intervals.

^d^Statistical significance assessed by comparing high- and low-risk areas within each period using Student’s t-test (all variables in lifestyle, medical status, medical accessibility, and health screening categories and the number of hospital beds in healthcare infrastructure) or the Mann–Whitney U test (all variables in demography, socioeconomic status, and physical environment categories and the number of medical personnels in healthcare infrastructure), with a Bonferroni-corrected significance threshold of p < 0.00217 (0.05/23).

^e^Number of districts

**References**

1. Cohen J. Statistical power analysis for the behavioral sciences. 2nd ed. Mahwah, NJ, USA: Lawrence Erlbaum Associates; 2013.
