# Supplementary material for: Spatio-temporal changes in clusters of gastric cancer incidence: The impact of nationwide cancer control programs in South Korea
Source: PLoS One. 2026 Jun 16;21(6):e0349384. doi: 10.1371/journal.pone.0349384 (PMC13271449; doi:10.1371/journal.pone.0349384)
Supplement: S6 Table — (DOCX) [file pone.0349384.s009.docx]

**S6 Table.** Associations of geographic characteristics and age-standardized gastric cancer incidence rates in 2009–2013 and 2014–2018, and their differences between two periods across 243 districts

| **District-level characteristics** | **2009–2013** | | **2014–2018** | | **Difference^b^** | |
| --- | --- | --- | --- | --- | --- | --- |
|  | **GC incidence change**  **(95% CI)^a^** | **P-value** | **GC incidence change**  **(95% CI)^a^** | **P-value** | **GC incidence change**  **(95% CI)** | **P-value** |
| Demography |  |  |  |  |  |  |
| % of older adults ≥ 65 years | 0.431 (0.271, 0.591) | <0.001 | 0.446 (0.331, 0.561) | <0.001 | 0.807 (0.067, 1.546) | 0.033 |
| Sex ratio^b^ | -0.106 (-0.410, 0.199) | 0.495 | 0.025 (-0.178, 0.228) | 0.806 | 0.691 (0.109, 1.274) | 0.020 |
| Population density | -0.051 (-0.069, -0.033) | <0.001 | -0.047 (-0.061, -0.032) | <0.001 | -0.015 (-0.238, 0.208) | 0.896 |
| % of urban-dwelling population | -0.111 (-0.153, -0.068) | <0.001 | -0.123 (-0.157, -0.089) | <0.001 | -0.133 (-0.293, 0.028) | 0.105 |
| Socio-economic status |  |  |  |  |  |  |
| Growth regional domestic product per capita (1,000 USD/person) | -0.004 (-0.069, 0.062) | 0.912 | -0.016 (-0.059, 0.028) | 0.479 | -0.092 (-0.237, 0.053) | 0.212 |
| % of higher education | -0.370 (-0.478, -0.262) | <0.001 | -0.402 (-0.483, -0.321) | <0.001 | -0.519 (-0.978, -0.061) | 0.027 |
| Lifestyle |  |  |  |  |  |  |
| % of breakfast ≥5 times/week | 0.585 (0.362, 0.808) | <0.001 | 0.367 (0.197, 0.538) | <0.001 | 0.189 (0.018, 0.360) | 0.030 |
| % of low-salt preference | 0.198 (-0.258, 0.653) | 0.393 | -0.257 (-0.660, 0.147) | 0.212 | 0.442 (0.145, 0.738) | 0.004 |
| % of current smoking | 0.326 (-0.135, 0.788) | 0.165 | 0.342 (-0.021, 0.706) | 0.065 | -0.230 (-0.557, 0.096) | 0.166 |
| % of heavy drinking | -0.228 (-0.533, 0.077) | 0.143 | 0.207 (-0.084, 0.497) | 0.162 | -0.142 (-0.339, 0.055) | 0.157 |
| % of moderate to vigorous physical activity | 0.058 (-0.078, 0.195) | 0.402 | 0.137 (-0.001, 0.274) | 0.051 | -0.069 (-0.160, 0.023) | 0.141 |
| % of regular walking | -0.135 (-0.236, -0.034) | 0.009 | -0.193 (-0.274, -0.112) | <0.001 | -0.047 (-0.115, 0.022) | 0.182 |
| % of self-reported obesity | -0.484 (-0.935, -0.032) | 0.036 | 0.344 (-0.021, 0.709) | 0.064 | -0.183 (-0.482, 0.115) | 0.228 |
| Medical status |  |  |  |  |  |  |
| % of doctor’s diagnosis of hypertension | -1.309 (-1.972, -0.646) | <0.001 | 0.118 (-0.416, 0.653) | 0.663 | -0.111 (-0.573, 0.350) | 0.636 |
| % of doctor’s diagnosis of diabetes | -1.096 (-2.401, 0.209) | 0.099 | 0.725 (-0.308, 1.758) | 0.168 | -0.011 (-0.690, 0.669) | 0.976 |
| % of doctor’s diagnosis of dyslipidemia | -1.004 (-1.517, -0.491) | <0.001 | -0.691 (-1.109, -0.272) | 0.001 | 0.101 (-0.257, 0.460) | 0.577 |
| Healthcare infrastructure |  |  |  |  |  |  |
| Number of hospital beds per 1000 people | 0.299 (0.119, 0.478) | 0.001 | 0.095 (-0.016, 0.205) | 0.092 | -0.041 (-0.272, 0.191) | 0.730 |
| Number of medical personnel per 1000 people | -0.493 (-1.110, 0.124) | 0.117 | -0.533 (-0.974, -0.092) | 0.018 | -0.910 (-3.329, 1.509) | 0.459 |
| Medical accessibility |  |  |  |  |  |  |
| % of unmet healthcare needs | -0.057 (-0.352, 0.239) | 0.705 | 0.206 (-0.056, 0.469) | 0.123 | -0.111 (-0.287, 0.065) | 0.217 |
| Health screening |  |  |  |  |  |  |
| % of cancer screening examinees for the previous 2 years | -0.104 (-0.370, 0.162) | 0.443 | -0.105 (-0.283, 0.074) | 0.249 | 0.121 (-0.016, 0.259) | 0.084 |
| % of gastric cancer screening examinees | 0.786 (0.514, 1.058) | <0.001 | 0.597 (0.389, 0.804) | <0.001 | 0.185 (-0.132, 0.501) | 0.252 |
| % of health screening examinees for the previous 2 years | -0.178 (-0.430, 0.074) | 0.166 | -0.240 (-0.454, -0.027) | 0.028 | 0.031 (-0.140, 0.201) | 0.723 |
| Physical environments |  |  |  |  |  |  |
| % of urban forest coverage within residential area | -0.132 (-0.325, 0.060) | 0.176 | -0.169 (-0.291, -0.047) | 0.007 | -0.020 (-0.213, 0.172) | 0.836 |

^a^Regression coefficients and 95% confidence intervals from district-level univariable linear regression models of age-standardized gastric cancer incidence rates (ASIR, per 100,000) on each characteristic. ^b^ASIR difference for 2009-2013 from 2014–2018.
