# Supplementary figures and images for: Spatio-temporal changes in clusters of gastric cancer incidence: The impact of nationwide cancer control programs in South Korea
Source: PLoS One. 2026 Jun 16;21(6):e0349384. doi: 10.1371/journal.pone.0349384 (PMC13271449; doi:10.1371/journal.pone.0349384)

**S1 Fig.** Global Moran’s I statistics against the number of neighboring districts

| 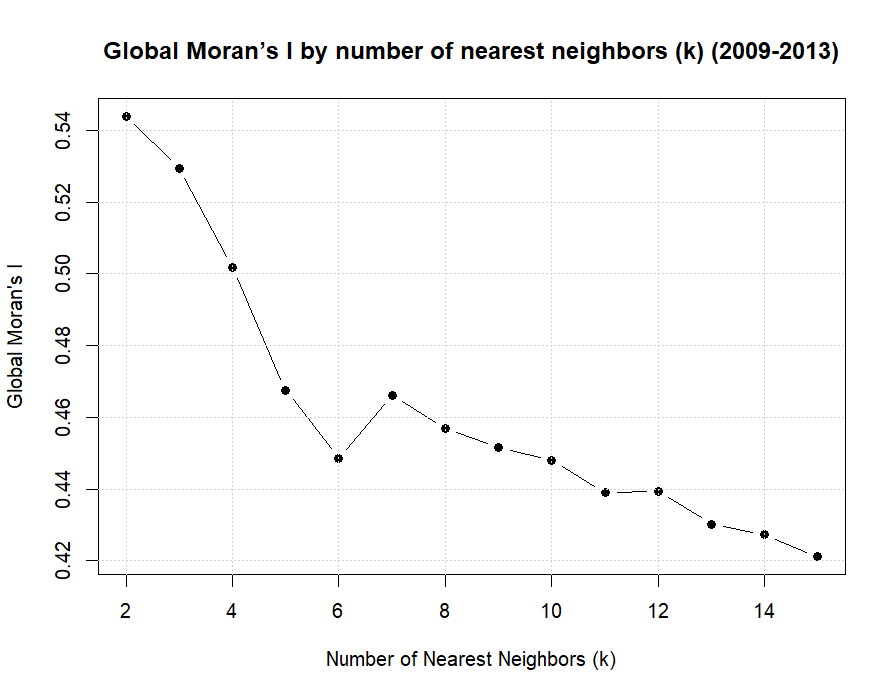 |
| --- |
| 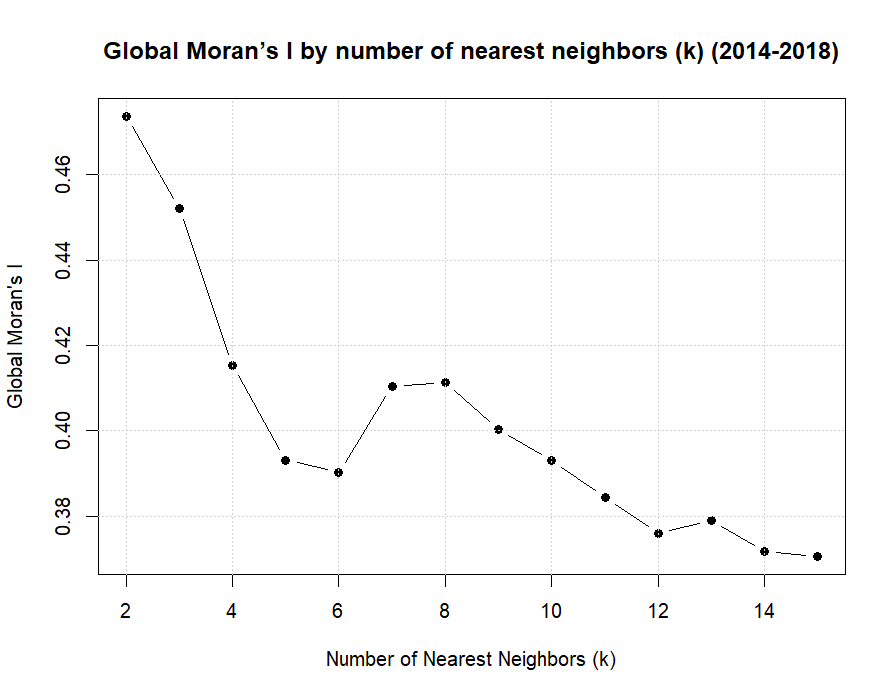 |

Supplement: S1 Fig — (DOCX) [file pone.0349384.s010.docx]
