# Supplementary material for: Spatio-temporal changes in clusters of gastric cancer incidence: The impact of nationwide cancer control programs in South Korea
Source: PLoS One. 2026 Jun 16;21(6):e0349384. doi: 10.1371/journal.pone.0349384 (PMC13271449; doi:10.1371/journal.pone.0349384)

**S2 Fig.** Maps of clustered districts based on **l**ocal Moran’s I (top) and Getis-Ord Gi (bottom) for age-standardized gastric cancer incidence rates in South Korea for 2009–2013 and 2014–2018

**
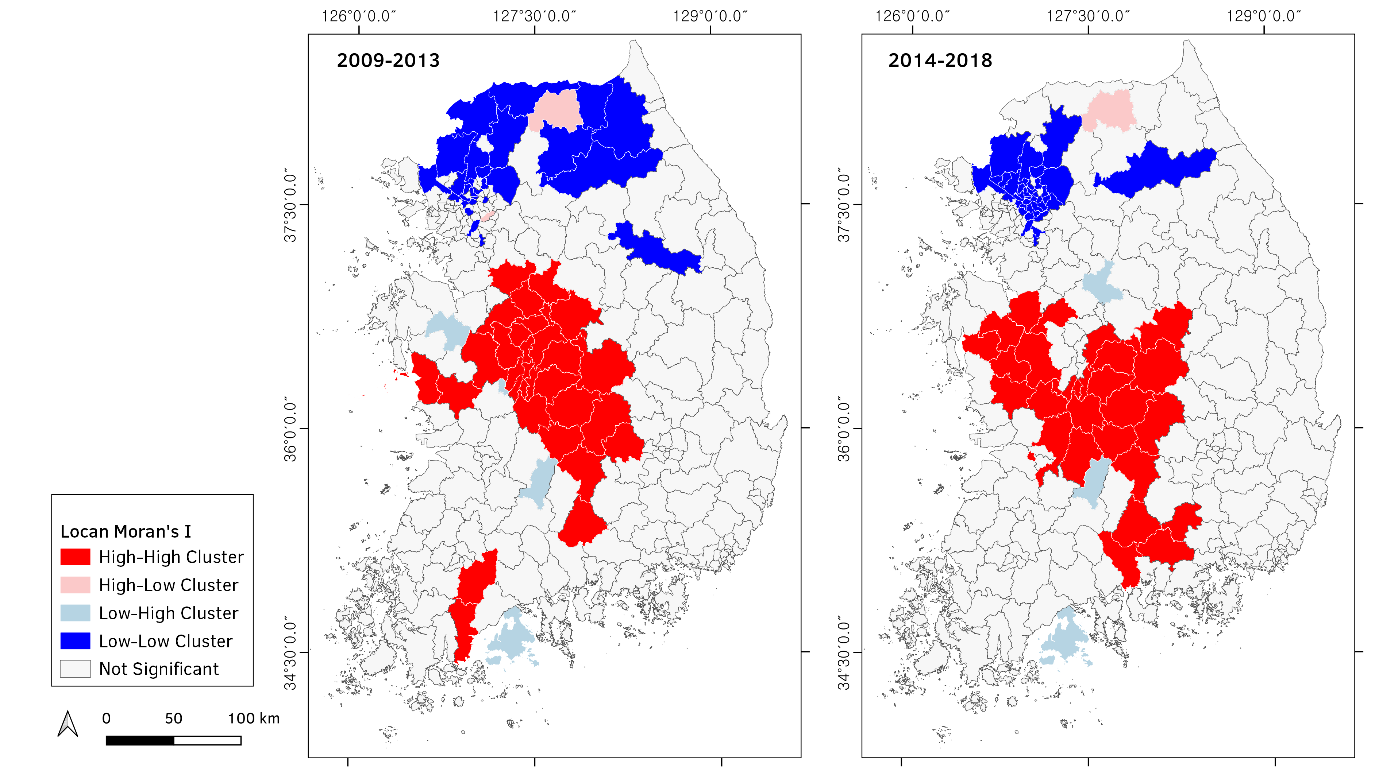
**


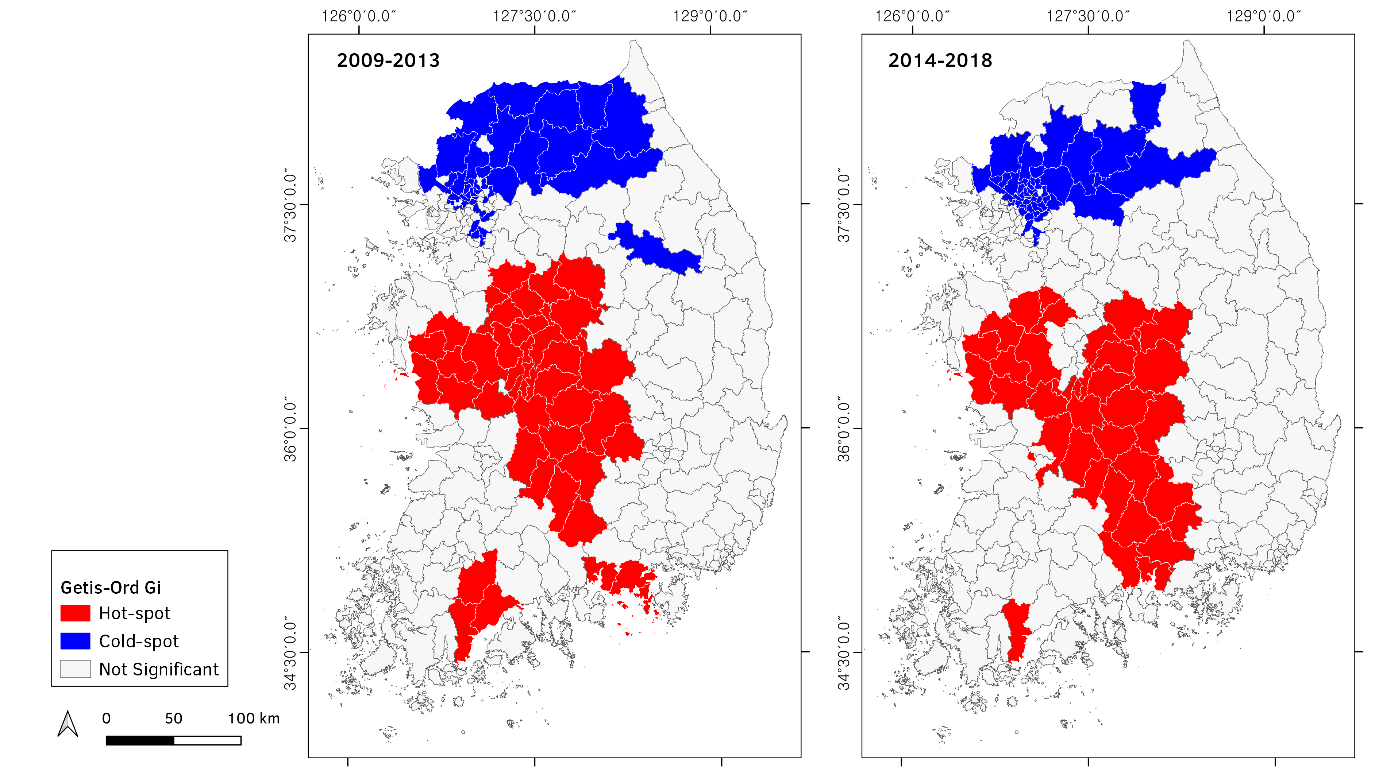

Supplement: S2 Fig — (DOCX) [file pone.0349384.s011.docx]
