# Supplementary material for: Spatio-temporal changes in clusters of gastric cancer incidence: The impact of nationwide cancer control programs in South Korea
Source: PLoS One. 2026 Jun 16;21(6):e0349384. doi: 10.1371/journal.pone.0349384 (PMC13271449; doi:10.1371/journal.pone.0349384)
Supplement: S4 Fig — (DOCX) [file pone.0349384.s013.docx]

**S4 Fig.** Maps of high- and low-risk areas of age-standardized incidence rates for gastric cancer across 243 districts in South Korea for 2009–2013 and 2014–2018 with progressive application of permutation test and/or multiplicity adjustment

| 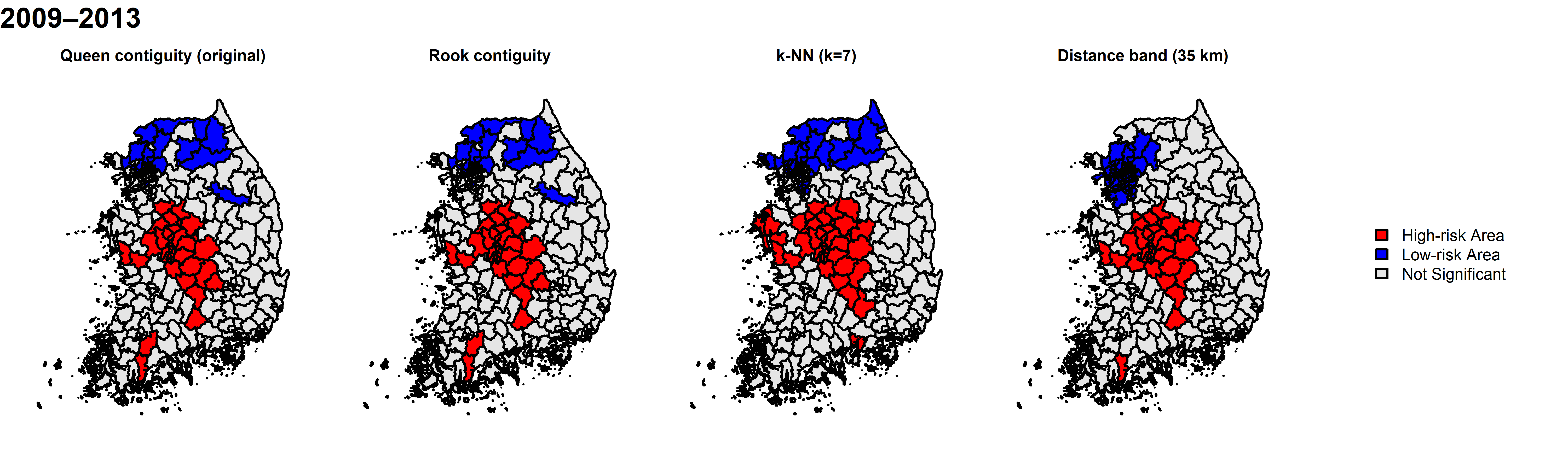 |
| --- |
| 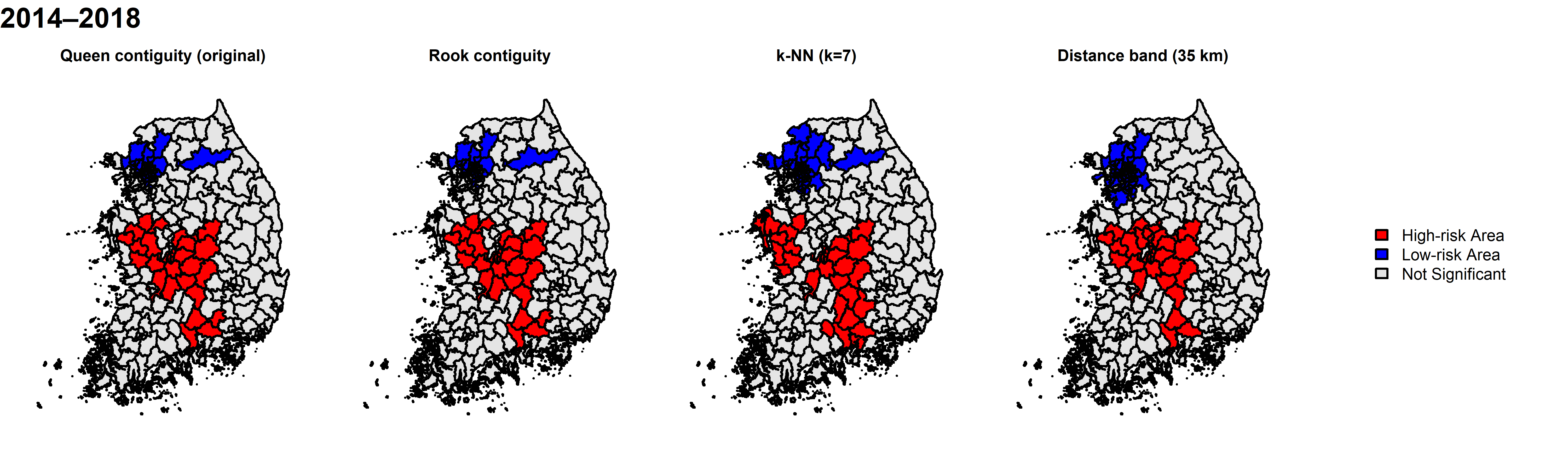 |
