# Supplementary material for: Spatio-temporal changes in clusters of gastric cancer incidence: The impact of nationwide cancer control programs in South Korea
Source: PLoS One. 2026 Jun 16;21(6):e0349384. doi: 10.1371/journal.pone.0349384 (PMC13271449; doi:10.1371/journal.pone.0349384)
Supplement: S5 Fig — (DOCX) [file pone.0349384.s014.docx]

**S5 Fig.** Box plots of four geographic characteristics between two periods (2009–2013 and 2014–2018) in the districts whereby risk clusters changed (high to non-significant (NS), NS to low, low to NS, and NS to high-risk areas) for age-standardized gastric cancer incidence rates

| 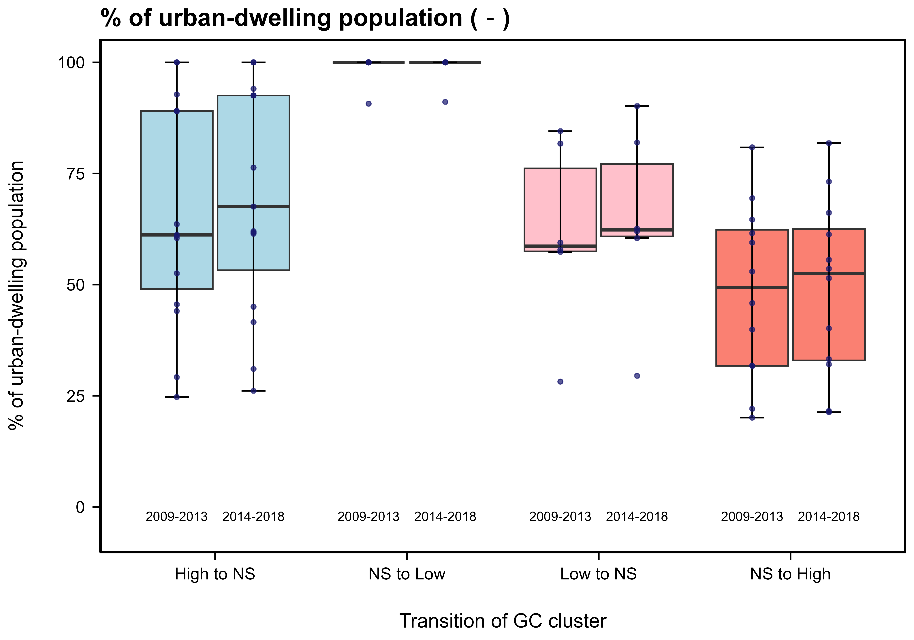 | 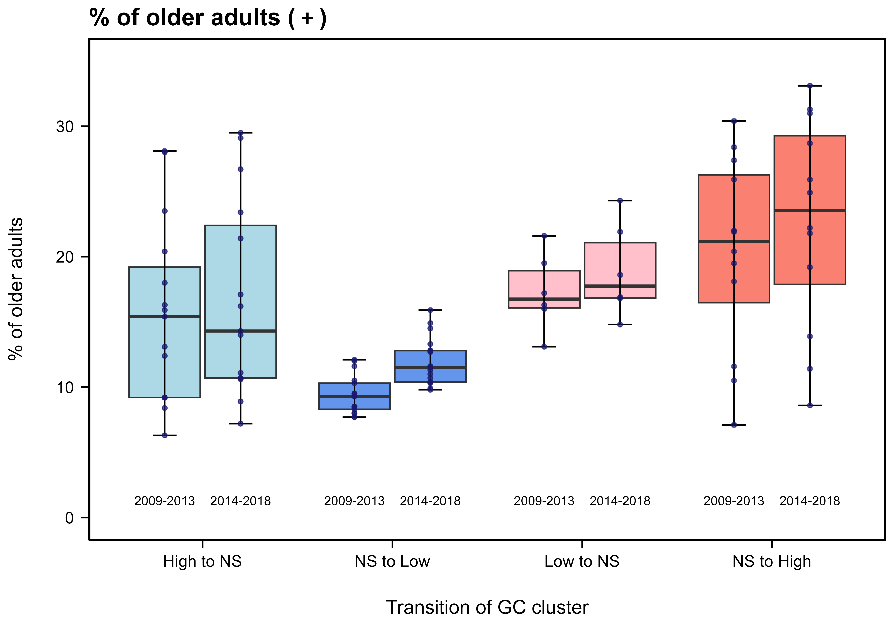 |
| --- | --- |
| 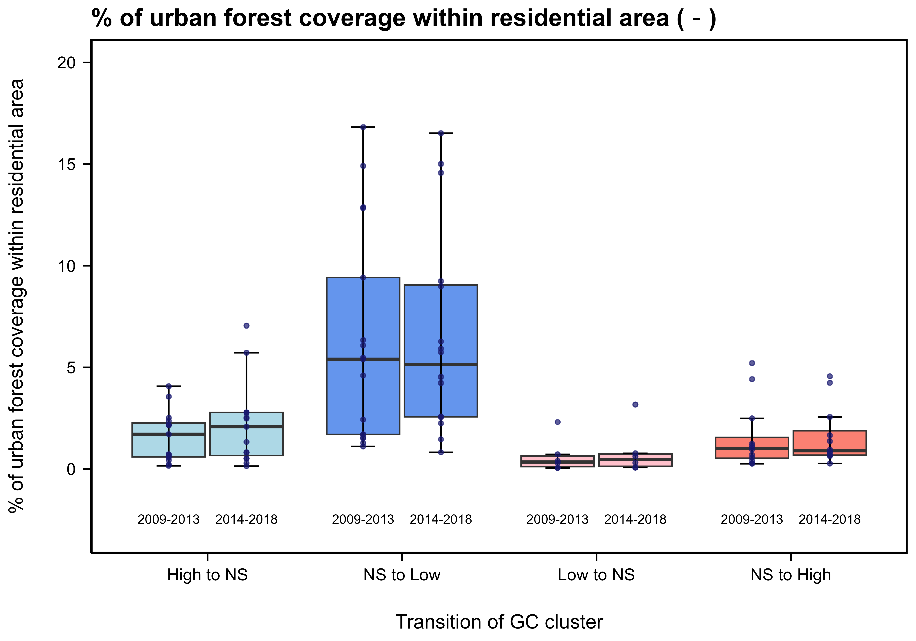 | 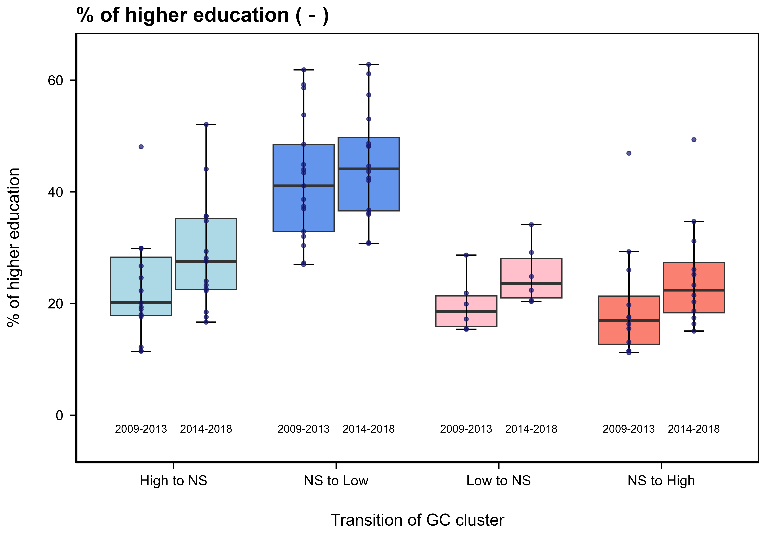 |
