# Supplementary material for: Spatio-temporal changes in clusters of gastric cancer incidence: The impact of nationwide cancer control programs in South Korea
Source: PLoS One. 2026 Jun 16;21(6):e0349384. doi: 10.1371/journal.pone.0349384 (PMC13271449; doi:10.1371/journal.pone.0349384)
Supplement: S6 Fig — (DOCX) [file pone.0349384.s015.docx]

**S6 Fig.** Map of the districts where clusters changed (high to non-significant (NS), NS to low, low to NS, and NS to high-risk areas) for age-standardized gastric cancer incidence rates

**
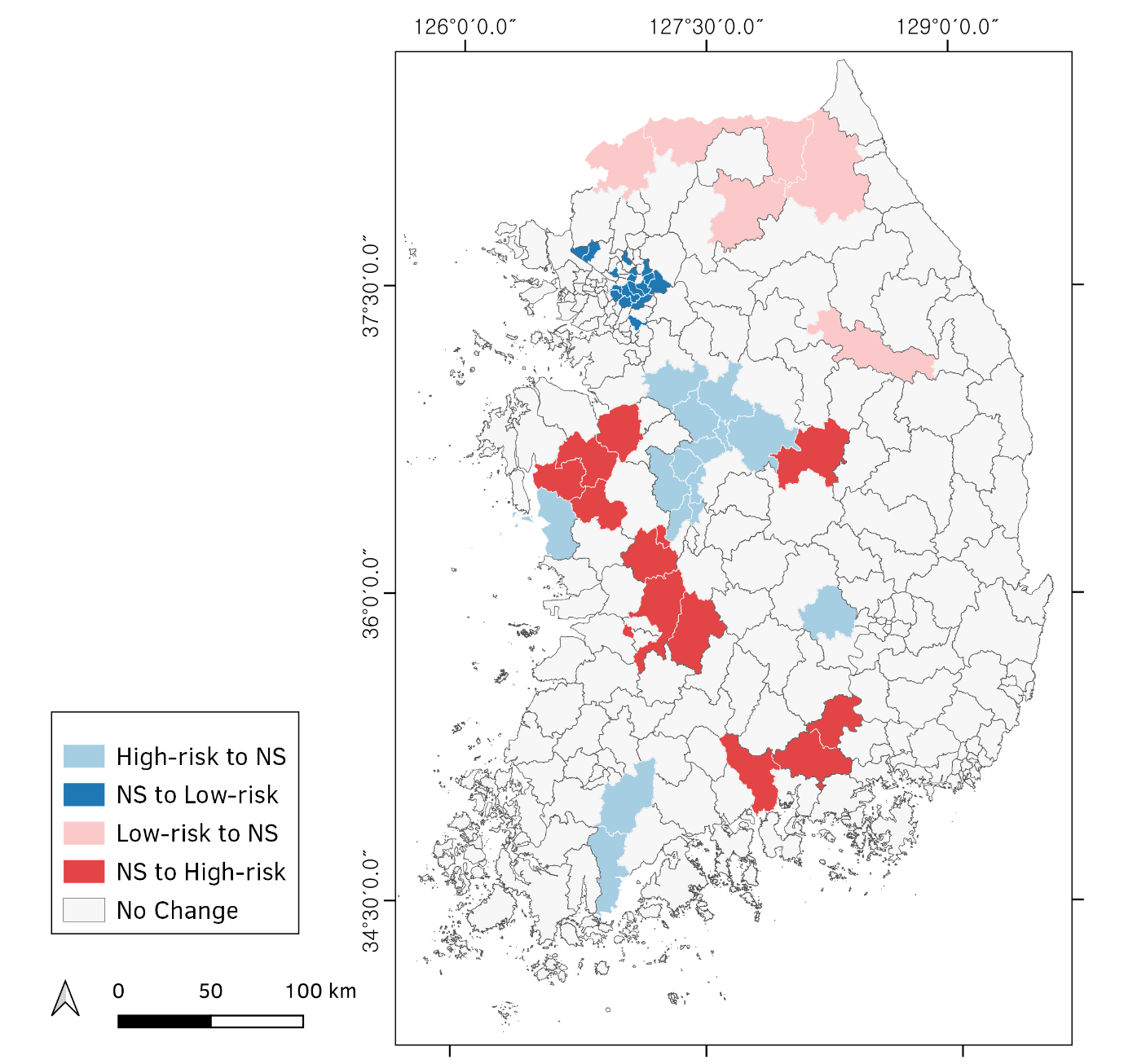
**
