# Supplementary material for: Spatio-temporal changes in clusters of gastric cancer incidence: The impact of nationwide cancer control programs in South Korea
Source: PLoS One. 2026 Jun 16;21(6):e0349384. doi: 10.1371/journal.pone.0349384 (PMC13271449; doi:10.1371/journal.pone.0349384)
Supplement: S7 Fig — (DOCX) [file pone.0349384.s016.docx]

**S7 Fig.** Maps of gastric cancer risk clusters across 243 districts and three provinces with high *H. pylori* eradication in South Korea by 2009–2013 and 2014–2018

| 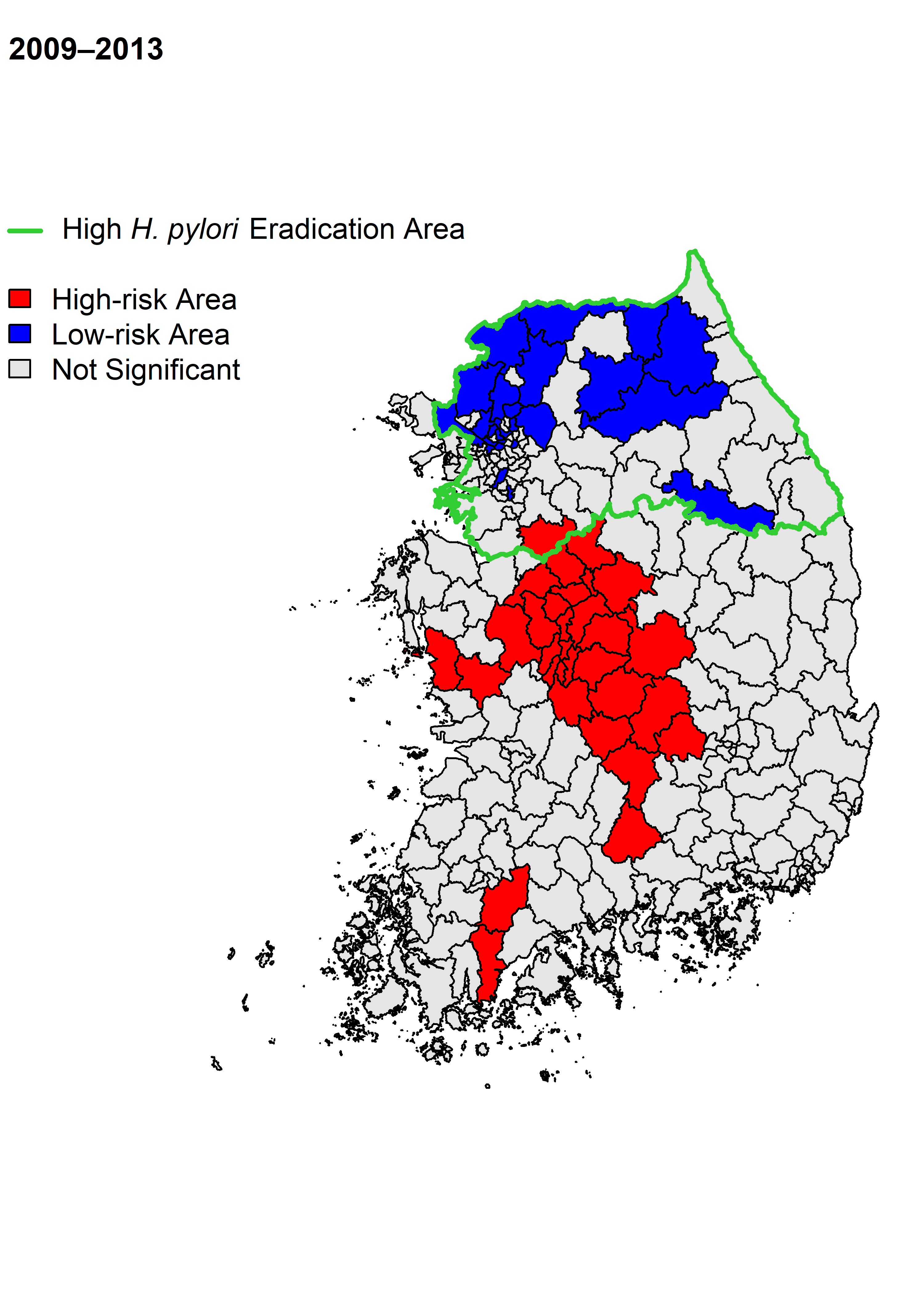 | 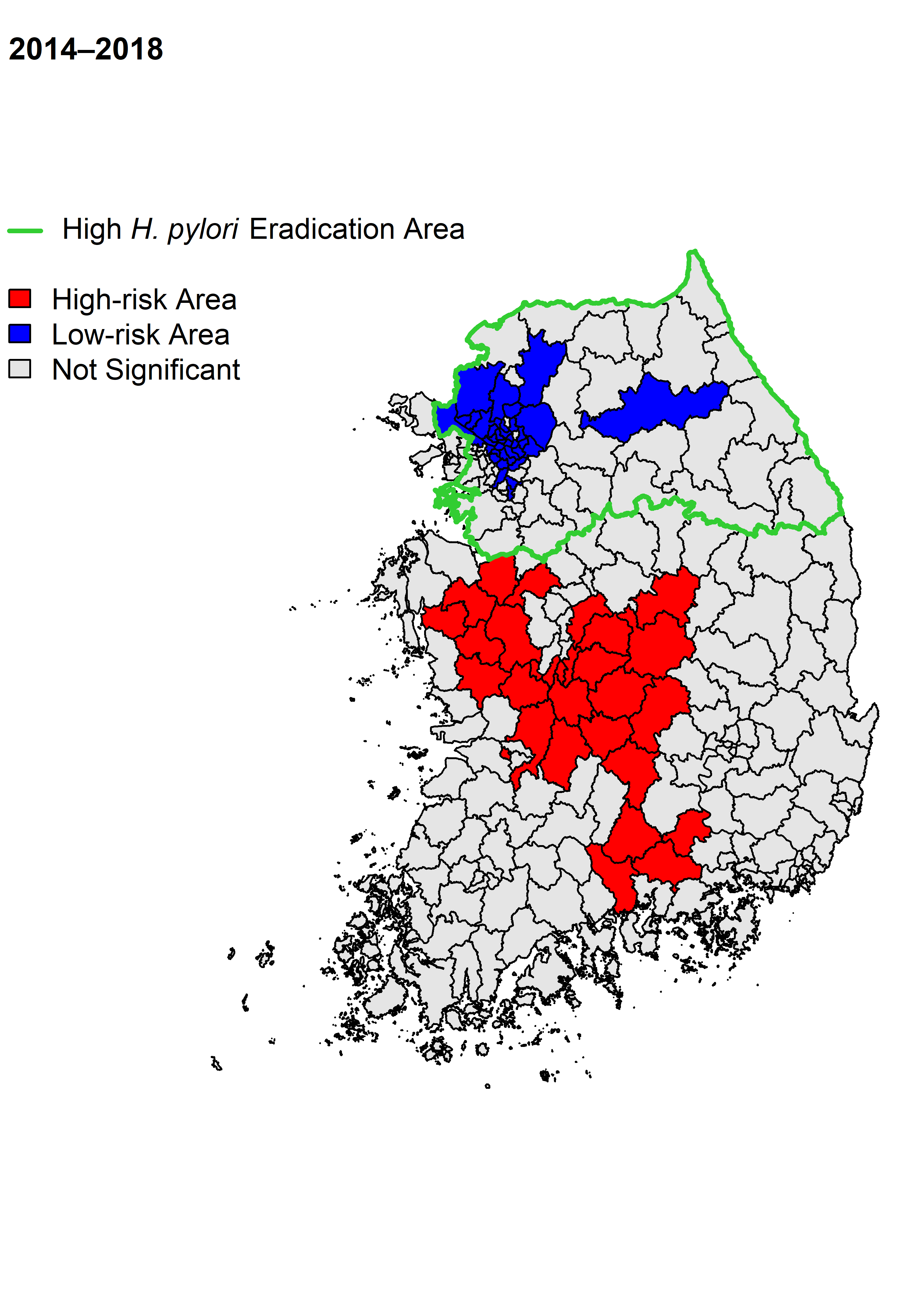 |
| --- | --- |
